# A High‐Current‐Tolerant Multimetallic Phosphide Electrode for Alkaline Water Electrolysis Toward Industrial Conditions

**DOI:** 10.1002/advs.76083

**Published:** 2026-06-12

**Authors:** Yang Li, Chunling Lu, Biao Wang, Dongchao Qiu, Bingbing Niu, Tao Feng

**Affiliations:** ^1^ School of Science University of Science and Technology Liaoning Anshan China; ^2^ Key Laboratory of Interfacial Physics and Technology Shanghai Institute of Applied Physics Chinese Academy of Sciences Shanghai China; ^3^ Department of Materials Science and Engineering Southern University of Science and Technology Shenzhen China

**Keywords:** alkaline water electrolysis, high‐current‐tolerant, low‐noble‐metal dependency, multimetallic phosphide electrocatalyst

## Abstract

The development of electrocatalysts that maintain high efficiency and durability at industrial current densities remains a pivotal challenge for alkaline water electrolysis. Here, we present a high‐performance phosphide‐modified multimetallic hydrogen evolution reaction (HER) catalyst, NiFe_0.33_RuO_x_@P, with high‐current‐tolerant and low noble‐metal content. In this catalyst, Ni and Fe mainly exist as phosphide‐derived species with partial surface oxidation, while Ru is predominantly present as RuP‐like active domains embedded in the multimetallic matrix. The electrolyzer with a Ni foam anode and NiFe_0.33_RuO_x_@P cathode delivers an exceptional current density of 1.7 A cm^−2^ at 2.0 V under 6 m KOH (≈ 30%) at 70°C, demonstrating outstanding industrial‐level performance. This catalyst requires an overpotential of only 72 mV to achieve 100 mA cm^−2^ for HER in 1 M KOH, lower than commercial Pt/C. Critically, it sustains stable operation for over 200 h in 6.0 m KOH at 500 mA cm^−2^. Scalability is confirmed on a 25 cm^2^ electrode, underscoring its practical potential. The exceptional stability and activity are rooted in a phosphorus‐tuned electronic structure that yields a near‐optimal hydrogen adsorption free energy (ΔG_H*_ tuned from +0.498 to −0.153 eV).

## Graphical abstract

A phosphide‐modified multimetallic HER electrode achieves industrial‐level performance with minimal noble‐metal content. It delivers 1.7 A cm^−2^ at 2.0 V in 6 m KOH at 70°C and operates stably for over 200 h at 500 mA cm^−2^. Phosphorus tuning of the electronic structure optimizes hydrogen adsorption, offering a durable, high‐rate pathway for alkaline water electrolysis.

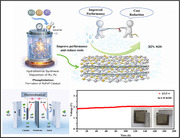

## 1 Introduction

Alkaline water electrolysis has been regarded as one of the most promising technological routes for the large‐scale commercial production of green hydrogen due to its mature system, relatively low cost, and ease of scalability [1]. However, achieving efficient hydrogen evolution reaction (HER) under industrially relevant conditions remains a major challenge. In particular, catalyst durability and performance degradation under high current density operation severely limit practical applicability [2]. Although Pt‐based catalysts exhibit near‐optimal activity, their high cost and scarcity hinder large‐scale deployment [3]. Therefore, developing HER catalysts that can simultaneously deliver high activity, long‐term stability, and robust performance at high current densities is essential for advancing practical alkaline water electrolysis technologies [4].

Ruthenium‐based catalysts have attracted significant attention due to their near‐optimal hydrogen adsorption energetics and relatively lower cost compared to Pt [5]. Meanwhile, Fe‐based components offer advantages in terms of earth abundance and electronic modulation capability, despite their limited intrinsic activity [6]. Constructing Ru‐Fe bimetallic systems has emerged as an effective strategy to combine the high activity of Ru with the electronic regulation effect of Fe, thereby optimizing reaction intermediates and enhancing HER kinetics. For example, He et al. demonstrated a supported RuFe/FeNC catalyst with ultralow overpotentials (9.3 mV vs RHE) and a high turnover frequency of 1.35 H_2_ s^−1^ at −0.025 V RHE, surpassing commercial 20 wt.% Pt/C benchmarks in alkaline media [7]. Similarly, Wang et al. synthesized Ru/FeN_x_/C catalysts, achieving an overpotential of just 15 mV at 10 mA cm^−2^ in 1.0  KOH, showing enhanced reaction kinetics due to the electronic interaction between Ru and FeN_x_ species [8]. However, despite these advances, several critical challenges remain unresolved. In particular, the performance of Ru‐Fe catalysts under industrially relevant high‐current conditions remains insufficiently understood, while their structural stability and surface evolution during long‐term operation are rarely considered. Therefore, developing Ru‐Fe‐based catalysts that can simultaneously achieve high‐current tolerance, structural robustness, and efficient noble‐metal utilization remains an urgent challenge, motivating the present work.

Phosphidation is widely recognized as an effective strategy to regulate the electronic structure and surface properties of transition‐metal catalysts [9]. It enables modulation of metal d‐electron density and optimization of hydrogen adsorption energetics [10]. However, despite the rapid development of Ru‐based phosphide catalysts, their catalytic behavior under realistic operating conditions remains far from fully understood. In particular, previous studies have suggested that phosphide surfaces may undergo partial oxidation or surface evolution under alkaline HER conditions, leading to the coexistence of phosphide and oxygen‐containing species [11]. More importantly, there remains a significant gap between laboratory‐scale evaluations and industrial alkaline electrolysis conditions for Ru‐based phosphide catalysts, which typically involve concentrated electrolytes (6 M KOH) and high current densities (≥ 500 mA cm^−2^) [12]. Catalysts that perform well under mild conditions often fail to maintain stability and activity under such harsh environments [13].

To address this gap, several critical challenges must be overcome. First, most reported catalysts are evaluated under relatively low current densities (< 100 mA cm^−2^), which are far from industrial requirements (≥ 500 mA cm^−2^) [12]. Second, their long‐term stability under concentrated alkaline electrolytes remains insufficiently demonstrated [12]. Third, performance validation at device level (e.g., membrane electrode assembly and large‐area electrodes) is rarely reported [14]. To address these challenges, we develop a phosphidation‐engineered Ni‐Fe‐Ru multimetallic electrode. This work aims to (i) design a high‐performance HER catalyst with excellent activity under industrially relevant conditions (6 M KOH and high current density), (ii) demonstrate long‐term durability under high‐current operation, and (iii) validate catalyst performance at device level using membrane electrode assembly and large‐area configurations.

## 2 Result and Discussion

**FIGURE 1:**
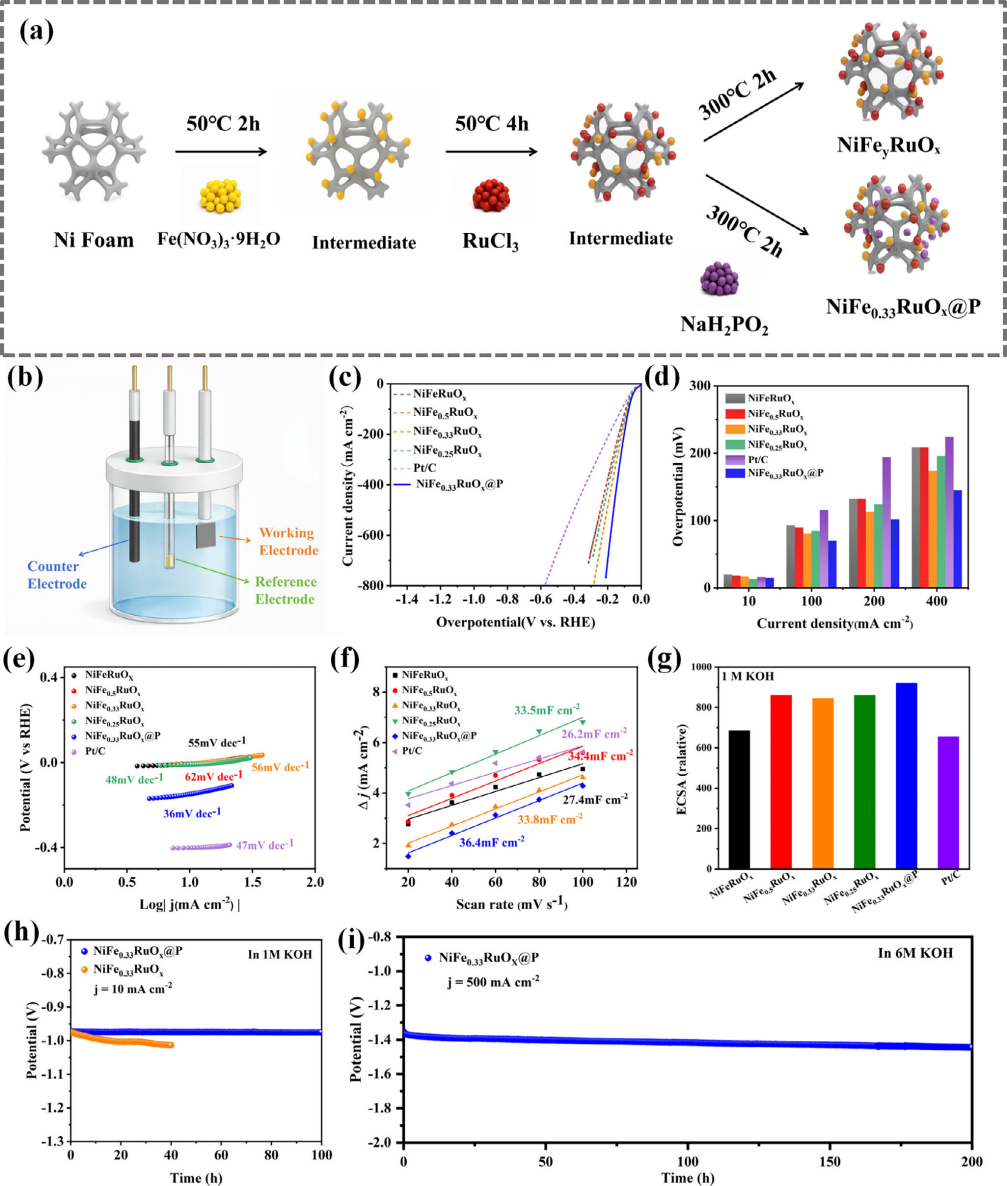
(a) Synthesis diagram of NiFe_y_RuO_x_ and NiFe_0.33_RuO_x_@P. (b) Schematic diagram of a three‐electrode setup. (c) Comparison of HER performance of NiFeyRuO_x_ and commercial Pt/C in 1.0 M KOH. (d) Overpotential comparison at 10, 100, 200, and 400 mA cm^−2^. (e) Tafel slope comparison. (f) C_dl_ comparison. (g) ECSA comparison. (h) Three‐electrode stability test for HER comparison of NiFe_0.33_RuO_x_@P for 100 h and NiFe_0.33_RuO_x_ for 50 h at −10 mA cm^−2^. (i) Three‐electrode stability test for HER of NiFe_0.33_RuO_x_@P at −500 mA cm^−2^ for 200 h.

Figure 1a illustrates the synthesis process of the catalyst, with the detailed synthetic procedure provided in the Experimental section. A three‐electrode configuration was used to evaluate the HER electrochemical performance of the samples, as schematically illustrated in Figure 1b. Linear sweep voltammetry (LSV) measurements were performed in 1.0 M KOH to evaluate the HER activity of the catalysts, as shown in Figure 1c. Among all samples, NiFe_0.33_RuO_x_@P exhibits the highest electrocatalytic activity. Compared with the unphosphorized NiFeRuO_x_ (81 mV), NiFe_0.33_RuO_x_@P requires substantially lower overpotentials to reach −100 mA cm^−2^ (72 mV). To quantitatively compare the catalytic kinetics over a wide current density range, the overpotentials at representative current densities were extracted and summarized in Figure 1d. At 10, 100, 200, and 400 mA cm^−2^, NiFe_0.33_RuO_x_@P consistently exhibits the lowest overpotentials among all catalysts, maintaining a clear advantage over Pt/C. At 100 mA cm^−2^, NiFe_0.33_RuO_x_@P requires an overpotential of approximately 72 mV, compared to ∼120 mV for Pt/C; at 200 mA cm^−2^, the corresponding overpotentials increase to ∼100 and ∼190 mV, respectively. Even at an industrially relevant current density of 400 mA cm^−2^, NiFe_0.33_RuO_x_@P preserves an overpotential advantage exceeding 60 mV than Pt/C, demonstrating its superior HER kinetics across the entire current density range.

As shown in Figure 1e, the NiFeRuO_x_‐based electrocatalysts exhibit distinctly different Tafel slopes under alkaline conditions. Among them, NiFe_0.33_RuO_x_@P delivers the lowest Tafel slope of 36 mV·dec^−1^, which is significantly smaller than those of NiFe_0.25_RuO_x_ (48 mV·dec^−1^), NiFe_0.33_RuO_x_ (55 mV·dec^−1^), and NiFe_0.5_RuO_x_ (62 mV·dec^−1^), as well as the commercial Pt/C catalyst (47 mV·dec^−1^). The reduced Tafel slope indicates accelerated reaction kinetics and more efficient electron transfer during the HER. According to classical Tafel analysis, a slope value in the range of ∼30–40 mV·dec^−1^ generally suggests that the HER kinetics tend to follow a Volmer–Tafel‐type pathway, in which the initial hydrogen adsorption (Volmer step) is relatively fast, while the subsequent surface hydrogen recombination step is likely involved in or close to the rate‐determining process [15, 16].

To further evaluate the electrochemically active surface area (ECSA), the double‐layer capacitance (C_dl_) was extracted from cyclic voltammetry (CV) curves recorded at different scan rates within the non‐Faradaic potential region across scan rates ranging from 10 to 120 mV/s (Figure S1a–f). The current density difference at each scan rate was calculated from CV profiles, with the linear slope of Δj versus scan rate providing a quantitative estimation of the C_dl_. Here, *Δj* is obtained as half of the difference between anodic and cathodic current densities and represents the capacitive charging current of the electrochemical double layer.

(1) $$\Delta j\;=\frac{j_{\mathrm{anode}}-\;j_{\mathrm{cathode}}}{2}$$

As shown in Figure 1f, NiFe_0.33_RuO_X_@P exhibits the highest C_dl_ value of 36.4 mF·cm^−2^, which is markedly higher than those of NiFe_0.33_RuO_x_ (33.8 mF·cm^−2^) and Pt/C (26.2 mF·cm^−2^). The larger C_dl_ value indicates a higher density of exposed electrochemically active sites, facilitating interfacial charge accumulation and transfer during the catalytic process [17].

The HER performance of electrocatalysts is closely related to their ECSA. The ECSA of each electrocatalyst was calculated using the equation ECSA = C_dl_/C_s_, where C_s_ was taken as 40 µF·cm^−2^, the ECSA of each catalyst was further estimated, as summarized in Figure 1g. NiFe_0.33_RuO_x_@P delivers the largest ECSA among all investigated samples, confirming the presence of a significantly higher number of accessible active sites [18]. Combined with the Tafel slope analysis, these results demonstrate that the superior HER performance of NiFe_0.33_RuO_x_@P originates not only from enhanced intrinsic reaction kinetics but also from its enlarged electrochemically active surface area and improved utilization of active sites. To further assess the catalytic activity and noble‐metal utilization, the turnover frequency (TOF) was estimated following the reported method [19]:

(2) $$\mathrm{TOF}=\frac{j}{\mathrm{mFn}}$$

where *j* is the geometric current density at a given overpotential, F is Faraday's constant, and *m* = 2 for the HER (2 electrons to form one H_2_). The number of electrochemically addressable active sites (*n*, mol) can be obtained from the voltammetric charge Q as n = Q/(2F), which yields TOF = J/Q for the HER. However, for multicomponent systems such as NiFe_0.33_RuO_x_@P, the direct quantification of electrochemically accessible active sites is challenging due to the overlap of redox features and the difficulty in unambiguously assigning charge contributions to specific active species (e.g., Ni, Fe, or Ru). Therefore, in this work, an apparent TOF normalized to the noble‐metal (Ru) content is adopted [20, 21]. Specifically, n is approximated by the Ru molar amount per geometric area derived from ICP analysis (Ru in Figure 3g), expressed as: *n*_Ru_ = Γ_Ru_ /*M*_Ru_ (where Γ_Ru_ is the areal loading and *M*_Ru_ is the molar mass of Ru) [22]. This Ru‐normalized apparent TOF mainly serves as a descriptor for evaluating the utilization efficiency of noble‐metal sites, while not excluding the possible contribution of Ni and Fe species to the overall HER activity. Accordingly, at η = 100 mA cm^−2^, NiFe_0.33_RuO_x_@P (Γ_Ru_ = 0.5791 mg_Ru_ cm^−2^) yields n_Ru_ = 5.73 × 10^−6^ mol cm^−2^ and TOF_Ru_ = 9.04 × 10^−2^ s^−1^, whereas NiFe_0.33_RuO_x_ (Γ_Ru_ = 0.8665 mg_Ru_ cm^−2^) gives n_Ru_ = 8.57 × 10^−6^ mol cm^−2^ and TOF_Ru_ = 6.04 × 10^−2^ s^−1^. Notably, despite a 33.17% reduction in Ru loading after phosphorization, a 1.50‐fold increase in apparent TOF is achieved, indicating enhanced noble‐metal utilization efficiency (Figure S2).

As shown in Figure 1h,i, constant‐current stability tests were conducted for the NiFe_0.33_RuO_x_@P electrocatalyst in an alkaline electrolyte (1 m KOH) to evaluate its structural and electrochemical stability under different operating current densities. At a relatively low current density of 10 mA cm^−2^ (Figure 1h), the phosphorized sample exhibits excellent durability during 100 h of continuous operation, with the working potential showing only a slight decay from −0.973 to −0.975 V, corresponding to a voltage variation of approximately 0.2%. In contrast, the non‐phosphorized sample displays a more pronounced potential drift under the same testing conditions, indicating that phosphorization effectively suppresses the degradation of active phases during electrolysis. At a higher current density of 100 mA cm^−2^ (Figure S3), NiFe_0.33_RuO_x_@P maintains stable operation for 200 h, with the working potential increasing from −1.28 to −1.31 V, corresponding to a voltage decay of about 2.6%. At an industrially relevant current density of 500 mA cm^−2^ in 6 m KOH electrolyte (Figure 1i), NiFe_0.33_RuO_x_@P maintains stable operation for 200 h, with the working potential gradually increasing from −1.36 to −1.44 V, corresponding to a voltage decay of approximately 5.8%. Achieving such long‐term stability under the combined harsh conditions of high current density and concentrated alkaline electrolyte highlights the excellent structural robustness and electrochemical durability of the catalyst. It is well recognized that operation under these conditions (≥ 500 mA cm^−2^ and 6.0 m KOH) imposes significantly intensified challenges, including rapid gas evolution, severe bubble accumulation, increased interfacial stress, and dynamic surface reconstruction. Despite these demanding conditions, the NiFe_0.33_RuO_x_@P electrode maintains stable electrocatalytic performance over extended operation, demonstrating strong tolerance to high‐current stress as well as efficient mass transport and gas‐release capability. In addition, we have included a comprehensive comparison with representative state‐of‐the‐art alkaline HER catalysts reported in the literature to better highlight the advantages of our catalyst. Specifically, key performance metrics, including overpotential, Tafel slope, and long‐term stability, have been systematically summarized in Table S1. This comparison provides a clearer benchmarking against existing catalysts and significantly strengthens the demonstration of the advantages of our work. Together with its high catalytic activity, this result further confirms the strong practical potential of NiFe_0.33_RuO_x_@P as a cathode material for industrial alkaline water electrolysis.

In addition, a comparison of the stability before and after phosphorization clearly indicates that phosphorization significantly enhances the operational stability of the electrode under both low and high current density conditions. This improvement can be attributed to the formation of robust metal–nonmetal bonds induced by phosphorization [23].

**FIGURE 2:**
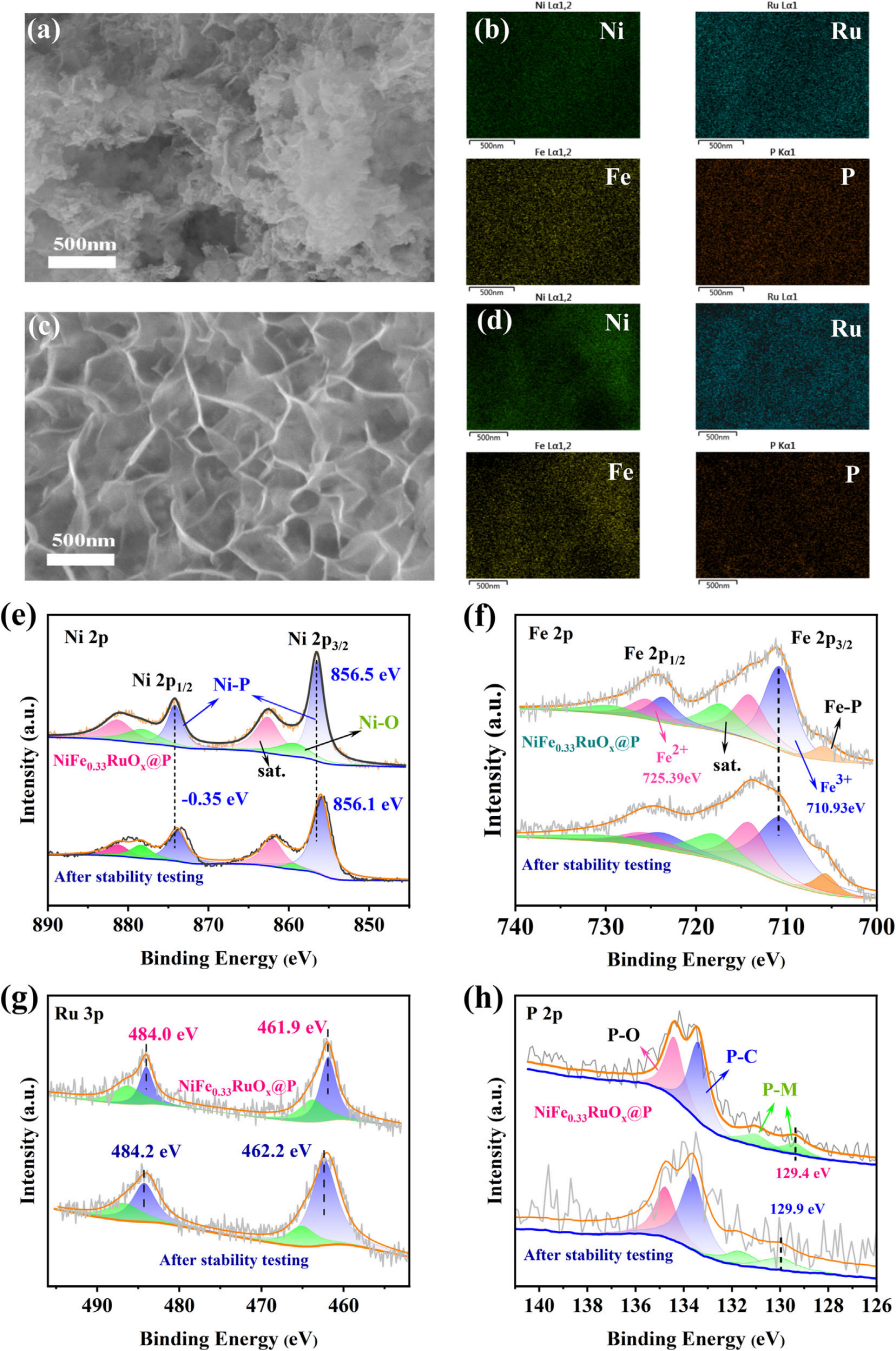
(a) SEM image of NiFe_0.33_RuO_x_@P. (b) The corresponding EDS mapping images. (c) SEM image of NiFe_0.33_RuO_x_@P after 200 h of stability testing. (d) The corresponding EDS mapping images. high‐resolution XPS spectra of (e)Ni 2p, (f) Fe 2p, (g) Ru 3p and (h) P 2p for NiFe_0.33_RuO_x_@P.

X‐ray diffraction (XRD) was employed to examine the crystalline characteristics of the samples synthesized via a hydrothermal route on nickel foam substrates. As shown in Figure S4, all diffraction patterns are dominated by three intense peaks located at approximately 44.5°, 51.8°, and 76.4°, which can be readily indexed to the (111), (200), and (220) planes of metallic Ni (PDF#00‐003‐1051, ICDD). No discernible diffraction peaks corresponding to NiFe_y_RuO_x_ or its phosphorized counterpart are observed. This phenomenon can be primarily ascribed to the overwhelming diffraction contribution from the nickel foam (NF) substrate. Owing to its high crystallinity, large thickness, and 3D porous architecture, NF generates strong diffraction signals that dominate the XRD patterns, thereby obscuring the relatively weak reflections originating from the surface‐grown active layers [24]. To further verify the structural characteristics, XRD measurements were additionally conducted on the powder sample of NiFe_0.33_RuO_x_@P (Figure S5). The diffraction pattern exhibits a broad and weak feature centered at ∼43°–45°, without any sharp diffraction peaks, indicating poor crystallinity and nanoscale domains. The significant peak broadening suggests small crystallite size and/or structural disorder [25]. Importantly, several weak diffraction contributions embedded within this broad feature are still consistent with the simulated RuP reference pattern, suggesting that the sample is not fully amorphous but contains RuP‐related short‐range ordered domains. To further investigate the morphology of NiFe_0.33_RuO_x_@P, scanning electron microscopy (SEM) was employed. As shown in Figure 2a, the NiFe_0.33_RuO_x_@P electrocatalyst exhibits a loosely stacked and porous morphology composed of irregular nanosheets/nanoparticles with a rough surface. Such a 3D porous structure has the potential to increase the specific surface area and expose abundant electrocatalytically active sites [26]. This porous and loosely stacked nanosheet morphology is closely correlated with the enhanced electrochemical surface properties of NiFe_0.33_RuO_x_@P. The enlarged catalyst/electrolyte interface provided by such a structure is expected to increase the ECSA and the number of accessible active sites, while also facilitating mass transport and gas evolution during HER. In addition, to clarify the effect of phosphorization on morphology, SEM characterization was also performed on the pristine NiFe_0.33_RuO_x_ sample. As shown in Figure S6, NiFe_0.33_RuO_x_ exhibits a relatively compact and partially agglomerated nanoparticle structure. Meanwhile, EDS mapping of NiFe_0.33_RuO_x_ (Figure S6b) shows that Ni, Fe, Ru, and O are uniformly distributed in the pristine sample. In comparison, for NiFe_0.33_RuO_x_@P (Figure 2b), the additionally introduced P element is also homogeneously distributed and spatially consistent with other elements, indicating successful incorporation of phosphorus and formation of a uniform multimetallic phosphide structure. The uniform elemental distribution is expected to induce synergistic effects among different metal species, which can effectively regulate the electronic structure and optimize the adsorption/desorption behavior of hydrogen intermediates [27]. Owing to the morphological evolution, NiFe_0.33_RuO_x_@P exhibits a higher ECSA (920) than that of NiFe_0.33_RuO_x_ (845). As shown in Figure 2c, after prolonged stability testing, the NiFe_0.33_RuO_x_@P electrocatalyst largely preserves its 3D porous structure composed of interconnected nanosheets. Although slight surface smoothing and restructuring can be observed, the overall porous framework remains intact without evident collapse or severe aggregation. The well‐preserved morphology indicates that the catalyst retains excellent mechanical integrity and structural stability under prolonged electrochemical operation. Such preserved porosity is crucial for maintaining efficient mass transport and continuous exposure of active sites during long‐term HER conditions, thereby accounting for the stable electrochemical performance observed during durability tests. Furthermore, EDS mapping of the post‐stability sample (Figure 2d) reveals a homogeneous spatial distribution of Ni, Fe, Ru, and P throughout the catalyst matrix. No noticeable elemental segregation or depletion was found after the stability testing, indicating that the multimetallic active components and phosphorus species remain well retained. Taken together, the preserved porous architecture and homogeneous elemental distribution suggest that NiFe_0.33_RuO_x_@P maintains excellent structural and compositional stability during prolonged HER operation.

As shown in Figure S7a, the HRTEM image exhibits the co‐existence of well‐resolved lattice fringes and localized disordered contrast, indicating that the material consists of nanocrystalline domains embedded in a low‐order matrix. The enlarged region shows a distinct interplanar spacing of d = 0.209 nm, which can be indexed to the (210) plane of RuP, confirming the effective phosphidation of Ru species and the formation of crystalline RuP nanodomains after NaH_2_PO_2_‐assisted phosphorization at 300°C under N_2_ [28]. In addition, the limited coherence length of the fringes together with noticeable orientational variations suggests nanosized crystallites with abundant grain boundaries and defects, which may increase the density of structural boundaries and potentially accessible active sites. Figure S7b presents the corresponding STEM‐EDS mappings. The low‐magnification STEM image reveals an agglomerated sub‐micrometer structure assembled from finer nanoscale building blocks. Notably, Ru and Fe signals remain uniformly distributed within the particle contour, demonstrating that the stepwise hydrothermal reaction of Fe followed by Ru enables intimate nanoscale coupling of the two metal components and preserves spatial homogeneity during the subsequent phosphorization process without apparent macroscopic phase segregation. Meanwhile, the P signal follows the spatial distribution trend of Ru and Fe, further supporting the phosphidation of the metal species and the formation of a metal–phosphide‐related structure represented by RuP. The Ni signal mainly originates from the NF substrate and residual substrate fragments introduced during sample preparation, reflecting close interfacial contact between the active phase and the conductive scaffold. Notably, an appreciable O signal is observed, indicating the presence of oxygen‐containing species on the surface and in locally enriched regions, such as a surface oxidized layer or oxygen‐containing phosphate or phosphide derivatives. Because STEM–EDS mapping is mainly used to visualize spatial distributions and the absolute intensities are not directly comparable among different elements, the pronounced O signal does not contradict the crystalline RuP domains identified by HRTEM; rather, it suggests that RuP nanodomains coexist with oxygen‐containing phases within the material. The catalyst consists of RuP‐like domains embedded in a dynamically reconstructed phosphide/oxide matrix, rather than a well‐defined crystalline phase.

To elucidate the surface valence states of the NiFe_0.33_RuO_x_@P catalyst, X‐ray photoelectron spectroscopy (XPS) was performed. The XPS survey spectrum (Figure S8) confirms that the sample mainly consists of Ni, Fe, Ru, and P, accompanied by minor C and O signals. The C signal originates from the unavoidable carbon contamination, while the O signal is associated with slight surface oxidation/hydroxylation and oxygen‐containing functional groups, while the NiFe_0.33_RuO_x_@P remains predominantly a multimetal phosphide in composition [29]. After the long‐term stability test, the XPS survey spectrum remains essentially unchanged, with no additional elemental signals detected. This observation indicates that the overall chemical composition of NiFe_0.33_RuO_x_@P is well preserved during prolonged electrochemical operation, demonstrating good compositional stability. In the high‐resolution Ni 2p spectrum (Figure 2e), the peaks at 855.8/873.5 eV are assigned to Ni─O species, whereas the lower‐binding‐energy components at 853.2/870.4 eV correspond to Ni─P bonds [24, 30]; pronounced shake‐up satellites at 861.6 and 879.8 eV further indicate surface Ni^2+^ (oxide/oxyhydroxide) species. Notably, relative to commonly reported reference values, the Ni─O component exhibits a slight positive shift, while the Ni‐P‐related features remain essentially unchanged (≤ 0.2 eV), which is reasonably attributed to oxidation‐induced charge redistribution at the surface [30]. After stability testing, the Ni─O component shifts to lower binding energy by ∼0.35 eV and shows a slightly increased relative intensity. Rather than indicating a simple increase in Ni oxidation state, this behavior is more reasonably attributed to partial surface reconstruction and changes in the local coordination/electronic environment of Ni under HER conditions. The increased Ni‐O‐related intensity suggests the formation or enrichment of surface oxidized/hydroxylated Ni species, while the negative shift reflects modified electronic screening and interfacial charge redistribution [31, 32].

The high‐resolution Fe 2p spectrum (Figure 2f) shows Fe 2p_3/2_ and Fe 2p_1/2_ peaks at 711–713 eV and 724–726 eV with evident satellites, indicating mixed Fe^2+^/Fe^3+^ states; compared with typical Fe─O references, the envelope appears slightly shifted/broadened, consistent with partial surface oxidation, while the lower‐binding‐energy components confirm Fe─P bonding and the incorporation of Fe into the multimetal phosphide framework. After stability testing, the Fe 2p spectral envelope becomes slightly broadened with a modest increase in the Fe^3+^ contribution, suggesting moderate surface reconstruction. Nevertheless, the persistence of Fe‐P‐related components indicates that Fe remains incorporated within the multimetal phosphide lattice, evidencing good structural stability. As shown in Figure 2g, the Ru 3p XPS spectra recorded before and after the stability test display two well‐defined characteristic peaks located at approximately 484.0 and 462.0 eV. For the pristine NiFe_0.33_RuO_x_@P sample, the two Ru 3p peaks are centered at 484.0 and 461.9 eV, respectively. After the stability test, these peaks slightly shift to 484.2 and 462.2 eV. The observed small positive shift of approximately 0.2–0.3 eV suggests only a subtle modulation of the local electronic structure of Ru during prolonged testing, without inducing significant changes in the Ru chemical state or the formation of new Ru species. Such a slight shift is generally attributed to minor variations in the coordination environment or electronic density around Ru atoms under electrochemical conditions, rather than structural degradation. Moreover, the overall spectral features, including peak shape and fitting components, remain nearly unchanged after the stability test. No noticeable peak broadening or abnormal intensity variation is observed, confirming the excellent chemical and structural stability of Ru sites. These results clearly demonstrate that the Ru active centers in NiFe_0.33_RuO_x_@P are well preserved after stability testing, which is essential for sustaining long‐term catalytic performance. Furthermore, the P 2p spectrum (Figure 2h) can be deconvoluted into three components: the low‐binding‐energy doublet at 129.5/130.4 eV assigned to P‐M (M = Ni, Fe, Ru), providing direct evidence for the multimetal phosphide structure and showing negligible deviation (≤ 0.1 eV); the P‐O component at 134.1 eV is moderately positively shifted relative to commonly reported phosphate‐like P‐O features (0.7–0.9 eV), attributable to surface reconstruction a; and the P‐C feature at ∼133.2 eV suggests P‐C coupling/doping that facilitates electronic modulation and interfacial charge transfer [33]. After long‐term operation, the P‐M doublet remains essentially unchanged, confirming the preservation of the multimetal phosphide skeleton. In contrast, the relative intensity of the P‐O component increases slightly, reflecting surface oxidation/reconstruction during HER. Importantly, the retained P‐C feature indicates stable interfacial coupling between phosphide species and the carbon scaffold, which is beneficial for sustained charge transfer and catalytic durability [34].

To further clarify whether the catalyst undergoes bulk phase transformation during long‐term operation, post‐catalysis HRTEM and STEM‐EDS characterizations were performed. The post‐stability HRTEM image still shows clear lattice fringes with an interplanar spacing of approximately 0.207 nm, which is consistent with the RuP‐related lattice spacing and close to the lattice fringe previously assigned to RuP in the pristine sample, indicating that RuP‐related nanocrystalline domains are retained after prolonged HER operation (Figure S9). Meanwhile, the corresponding post‐catalysis STEM‐EDS elemental mappings show that Ni, Fe, Ru, P, and O remain relatively uniformly distributed, without obvious elemental segregation or large‐scale phase separation. Combined with the slightly increased P‐O intensity observed in XPS, these results suggest that the catalyst mainly undergoes mild surface reconstruction during long‐term HER. The multimetal phosphide framework therefore remains largely intact, which is consistent with the excellent long‐term stability observed.

The XPS and HRTEM results clarify the existence states of the constituent elements in NiFe_0.33_RuO_x_@P. Ni mainly exists as Ni‐P species coupled with partially oxidized Ni‐O/Ni^2+^ surface environments, while Fe is present as Fe‐P species accompanied by mixed Fe^2+^/Fe^3+^ states, indicating a phosphide‐derived but partially reconstructed surface. Ru mainly exists in phosphide‐related active domains, consistent with the RuP‐like nanodomains revealed by HRTEM. Correspondingly, the catalytic roles of the individual components can be assigned as follows: Ru serves as the primary HER‐active center by providing favorable H^*^ adsorption sites; Fe acts as an electronic modulator that tunes the local coordination and electronic structure of neighboring Ru/Ni sites; Ni mainly contributes to conductive support, structural stabilization, and interfacial electronic regulation; and P plays a central role in establishing metal‐P coupling and surface charge redistribution. These combined effects endow NiFe_0.33_RuO_x_@P with a dynamically stabilized multimetal phosphide/oxide interface that is highly favorable for alkaline HER.

**FIGURE 3:**
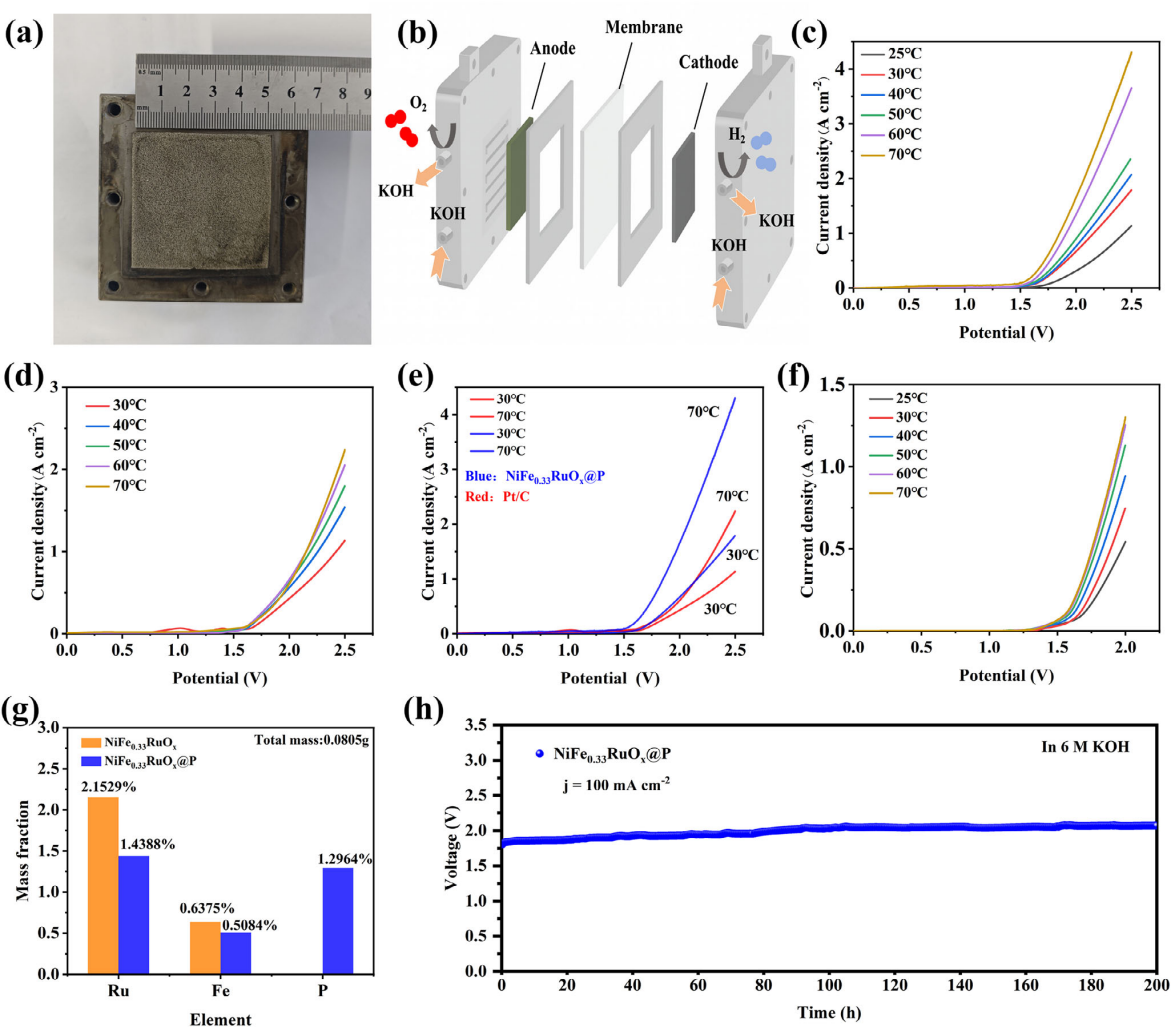
(a) Photograph of the MEA testing setup. (b) Membrane electrode assembly configuration. (c) Full cell of MEA (Ni foam (+) || NiFe_0.33_RuO_x_@P) LSV in 6 m KOH. (d) Full cell of MEA (Ni foam (+) || Pt/C) LSV in 6 m KOH. (e) Different temperature MEA LSV of Pt/C and NiFe_0.33_RuO_x_@P. (f) A 25 cm^2^ MEA (Ni foam (+) || NiFe_0.33_RuO_x_@P) LSV in 6 m KOH. (g) ICP of NiFe_0.33_RuO_x_ and NiFe_0.33_RuO_x_@P. (h) MEA stability test of NiFe_0.33_RuO_x_@P at 100 mA cm^−2^ for 200 h.

Figure 3a shows a photograph of the membrane electrode assembly (MEA) employed in this study, while Figure 3b displays the corresponding schematic diagram. All MEA measurements were conducted in 6 m KOH (≈ 30%), the commonly used electrolyte concentration for commercial alkaline water electrolysis [35]. NiFe_0.33_RuO_x_@P exhibits a consistent temperature‐promoted behavior from 25°C to 70°C: the polarization curves shift toward higher current densities with increasing temperature, higher current output at a given cell voltage (Figure 3c). This trend indicates that elevating temperature effectively reduces the overall polarization losses, with a more pronounced improvement in the high‐voltage (high‐load) regime, suggesting synergistic mitigation of coupled kinetic, ohmic, and mass‐transport limitations. Notably, 70°C lies within the typical mid‐to‐high operating window of industrial alkaline water electrolysis (commonly ∼60°C–80°C), highlighting the practical relevance of the MEA performance at this temperature. Meanwhile, the commercial Pt/C benchmark measured under identical MEA conditions also shows enhanced output at elevated temperature, providing a device‐level reference for comparison (Figure 3d).

A direct comparison between NiFe_0.33_RuO_x_@P and Pt/C was performed at 30°C and 70°C under the membrane electrode assembly configuration (Figure 3e). At a representative cell voltage of 2.0 V, Pt/C delivers current densities of approximately 0.35 A cm^−2^ at 30°C and 0.75 A cm^−2^ at 70°C, whereas NiFe_0.33_RuO_x_@P exhibits markedly higher values of about 0.70 and 1.70 A cm^−2^, respectively. Consequently, NiFe_0.33_RuO_x_@P achieves approximately 2.0‐fold and 2.3‐fold higher current densities than Pt/C at 30°C and 70°C, respectively, at 2.00 V. Moreover, upon increasing the operating temperature from 30°C to 70°C, the current density of NiFe_0.33_RuO_x_@P increases by approximately 2.4‐fold, exceeding the temperature‐induced enhancement observed for Pt/C (2.1‐fold). These results highlight the superior temperature responsiveness, high‐load adaptability, and enhanced current‐delivery capability of NiFe_0.33_RuO_x_@P under device‐relevant operating conditions. Upon increasing the electrode area from 2 to 25 cm^2^, the large‐area MEA still preserves the same temperature‐dependent polarization trend and delivers a high current density of ∼1.3 A cm^−2^ at 2.0 V, indicating promising scalability while also highlighting the need for further optimization of large‐area electrode architecture and assembly conditions (Figure 3f; Figure S10). Compared to small size electrode, the current density at 70°C and 2.0 V decreases from ∼1.7 to ∼1.3 A cm^−2^. This non‐negligible decline is likely associated with scale‐up‐induced device‐level losses, including less uniform current distribution, increased ohmic resistance, longer mass‐transport pathways, and more difficult bubble removal over the larger electrode area [36]. Nevertheless, to evaluate the change in Ru loading induced by phosphidation, ICP analysis was performed on the entire 2 cm^2^ electrode (catalyst deposited on Ni foam) after acid digestion (digested sample total mass, *m* = 0.0805 g) (Figure 3g). It should be noted that the reported mass includes both the active layer and the NF substrate. Therefore, the ICP results are not used to determine the absolute compositional weight fractions of the catalyst itself, but rather to compare the elemental loading before and after phosphidation. Based on the measured elemental content and the geometric electrode area (2 cm^2^), the Ru specific area loading before phosphidation was calculated to be 0.8665 mg_Ru_ cm^−2^, whereas that after phosphidation decreased to 0.5791 mg_Ru_ cm^−2^. This corresponds to a 33.17% decrease in Ru loading after phosphidation. Meanwhile, the Fe loading decreased from 0.2535 to 0.2044 mg_Fe_ cm^−2^. And phosphorus was successfully introduced, with an areal loading of 0.5218 mg_P_ cm^−2^. Since the same Ni foam substrate was used before and after phosphidation, its contribution remains constant, and thus the comparison of Ru and Fe loading variation is valid. These ICP results provide quantitative evidence that phosphidation reduces Ru usage while introducing P into the electrode, supporting the improved noble‐metal utilization observed in electrochemical measurements. For comparison, commercial Pt/C is commonly operated at a catalyst loading of 1 mg · cm^−2^. On this loading basis, NiFe_0.33_RuO_x_@P uses 0.5791 mg_Ru_ cm^−2^, corresponding to 57.9% of the total catalyst loading scale of Pt/C (1 mg · cm^−2^ Pt). Collectively, the ICP‐derived areal loadings provide reliable comparative evidence that phosphidation simultaneously introduces P and substantially decreases Ru usage, indicating improved noble‐metal utilization while remaining consistent with the maintained device‐level performance.

Regarding stability (Figure 3h), Chronopotentiometric testing was carried out at a constant current density of 100 mA cm^−2^ in 6 m KOH (≈ 30%) to evaluate the stability performance of NiFe_0.33_RuO_x_@P using an electrode with an active area of 25 cm^2^. The cell voltage exhibits a minor increase of approximately 1.2 mV/h during 200 h, reflecting sustained catalytic activity with minimal performance decay under demanding alkaline conditions.

**FIGURE 4:**
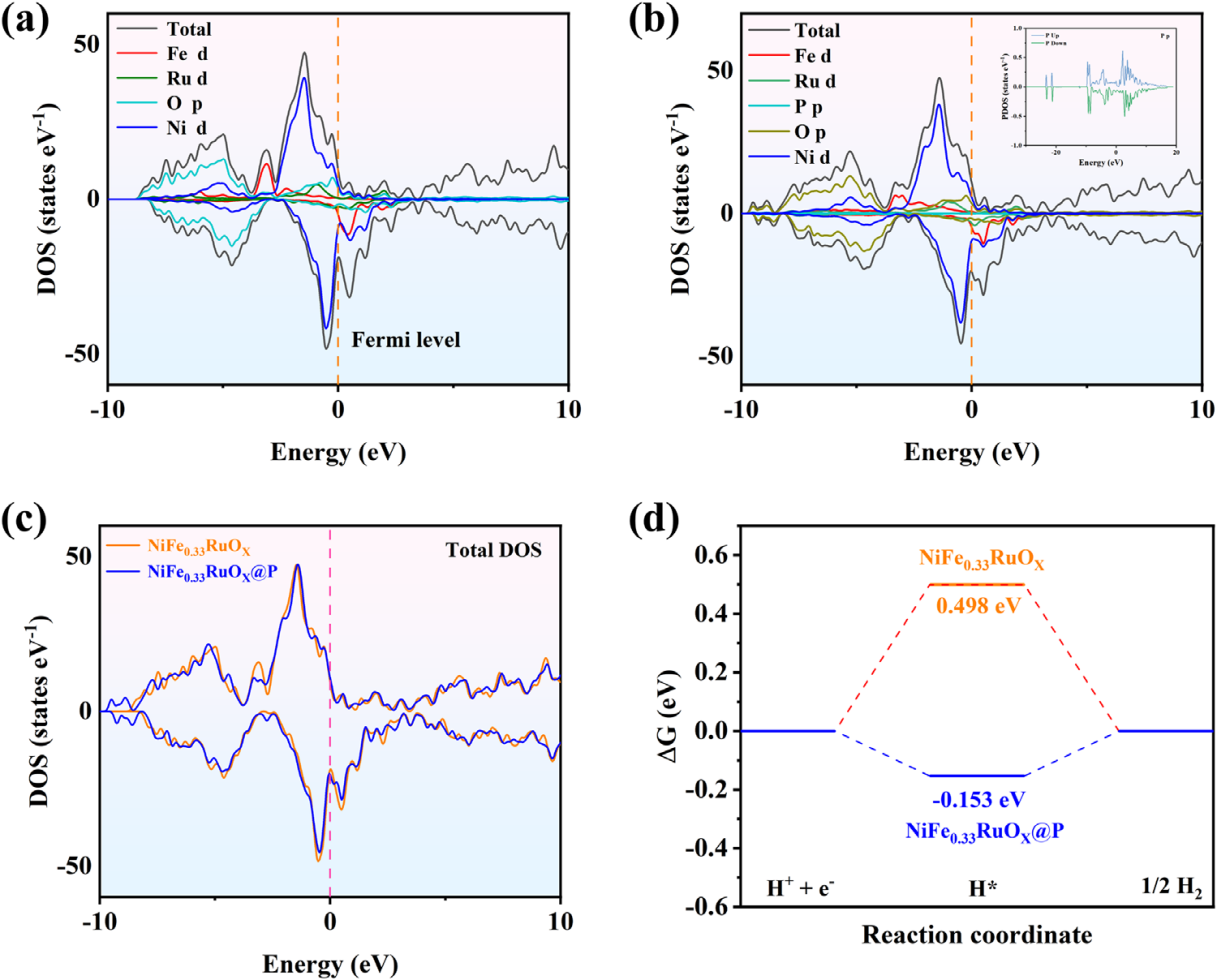
(a) The PDOS result of NiFe_0.33_RuO_x_. (b) The PDOS result of NiFe_0.33_RuO_x_@P. (c) Total DOS Comparison. (d) The HER free energy step diagram of NiFe_0.33_RuO_x_ and NiFe_0.33_RuO_x_@P.

Density functional theory (DFT) calculations were performed to elucidate the electronic‐structure evolution of NiFe_0.33_RuO_x_ upon phosphorization. Figures S11 and S12 present the structural model and the corresponding computational details, while Figure 4 summarizes the calculated density of states and HER thermodynamics [37]. For pristine NiFe_0.33_RuO_x_ (Figure 4a), the total density of states remains finite at the Fermi level, indicating metallic‐like electronic behavior that is favorable for charge transport [38]. The projected density of states further shows that the electronic states around *E_F_* are dominated mainly by Ni‐*d* orbitals, while Fe‐*d* and Ru‐*d* states provide secondary contributions. In contrast, the O‐*p* states are mainly distributed in the lower‐energy valence‐band region and exhibit relatively limited overlap with the transition‐metal *d* states near the Fermi level. These features indicate that the pristine NiFe_0.33_RuO_x_ possesses a predominantly metal‐centered near‐Fermi electronic structure, in which Ni‐derived *d* states play the major role in determining the electronic accessibility of the active surface [39]. After phosphorization, the electronic structure is noticeably modulated. As shown in Figure 4b, NiFe_0.33_RuO_x_@P still exhibits a finite DOS at the Fermi level, indicating preserved electronic conductivity. Compared with the pristine oxide, the phosphorized sample displays a more complex distribution of electronic states in the near‐*E_F_* region, in which Ni‐*d* states remain dominant, while Fe‐*d* and Ru‐*d* orbitals also contribute to the states around the Fermi level. By comparison, the direct contribution of P‐*p* states in this region is relatively limited. Nevertheless, the introduction of phosphorus still modifies the original metal‐centered electronic‐state distribution, indicating that phosphorization effectively tunes the local electronic environment of the transition‐metal active centers [40]. Such a redistribution of near‐Fermi‐level states is expected to improve the electronic accessibility of active sites and facilitate charge‐transfer processes relevant to alkaline HER, particularly the Volmer step associated with water dissociation [41, 42].

To more directly compare the global electronic response before and after phosphorization, the total DOS profiles are overlaid in Figure 4c. Both systems exhibit overall metallic characteristics, but an evident redistribution of electronic states is observed in the vicinity of the Fermi level after phosphorus incorporation. This comparison indicates that phosphorization does not simply increase the total number of states uniformly; rather, it reshapes the near‐*E_F_* electronic structure and alters the orbital composition around the active centers [43]. Such an electronic redistribution is consistent with the phosphorus‐induced modulation of the local coordination environment and provides an electronic‐structure basis for the altered adsorption behavior of HER intermediates.

The HER free‐energy diagram in Figure 4d further confirms the beneficial role of phosphorization in optimizing hydrogen adsorption. The pristine NiFe_0.33_RuO_x_ exhibits ΔG_H*_ = +0.498 eV, indicating thermodynamically unfavorable H^*^ adsorption due to overly weak hydrogen binding. After phosphorization, ΔG_H*_ decreases to −0.153 eV for NiFe_0.33_RuO_x_@P. Accordingly, |ΔG_H*_| is reduced from 0.498 to 0.153 eV, bringing the phosphorized catalyst much closer to the thermoneutral regime of ΔG_H*_ ≈ 0, which is generally regarded as favorable for HER [44]. This substantial optimization of hydrogen adsorption thermodynamics suggests that the phosphorized surface can more effectively balance H^*^ formation and H_2_ desorption, thereby providing a more favorable energetic pathway for hydrogen evolution.

## 3 Conclusion

In summary, we developed a P‐incorporated multimetallic alkaline HER catalyst, NiFe_0.33_RuO_x_@P, through phosphidation‐enabled electronic structure regulation, achieving simultaneously accelerated reaction kinetics and improved noble‐metal utilization. In this catalyst, Ni and Fe mainly exist as phosphide‐derived species with partial surface oxidation, while Ru is predominantly present as RuP‐like active domains embedded in the multimetallic matrix. Combined electrochemical measurements and DFT calculations, it was revealed that P incorporation induces charge redistribution and strengthens metal‐P electronic coupling, thereby optimizing hydrogen adsorption thermodynamics and facilitating faster HER kinetics. As a result, NiFe_0.33_RuO_x_@P exhibits high catalytic activity over a wide current‐density range in an alkaline electrolyte, together with favorable kinetic parameters and a large electrochemically active surface area. The catalyst further demonstrates excellent durability, maintaining stable performance during prolonged operation under both low and high current densities. Under device‐relevant membrane electrode assembly conditions, NiFe_0.33_RuO_x_@P consistently outperforms the benchmark Pt/C across a broad temperature window and sustains scalable current output on a large‐area electrode, while showing only a gradual voltage increase during long‐term full‐cell operation. Notably, the phosphidation strategy significantly reduces the Ru areal loading while preserving high‐current performance, highlighting the enhanced efficiency of noble‐metal utilization. Overall, this work establishes NiFe_0.33_RuO_x_@P as a highly active, high‐current‐tolerant, and durable alkaline HER catalyst, offering a promising design paradigm for practical and cost‐effective alkaline water electrolysis.

## 4 Materials and Methods

### 4.1 Materials

All chemicals used were of analytical grade and purchased from Aladdin. These include: Ruthenium chloride hydrate (RuCl_3_·xH_2_O, 99.9%), Iron nitrate (Fe(NO_3_)_3_·9H_2_O, 99.9%), hydrochloric acid (HCl, 1 mol L^−1^), Sodium hypophosphite monohydrate (NaH_2_PO_2_), and potassium hydroxide (KOH). Nickel foam (Ni foam, NF) was obtained from Shanghai Jiaqingyuan company. All reagents were used without further purification.

### 4.2 Synthesis of NiFe_y_RuO_x_

The experimental procedure is illustrated in Figure 1a. By fixing the molar amount of RuCl_3_·3H_2_O and using Fe(NO_3_)_3_·9H_2_O as the iron source, the molar ratios of Fe to Ru were adjusted to 1:1, 1:2, 1:3, and 1:4. Specifically, different amounts of Fe(NO_3_)_3_·9H_2_O were first dissolved in 10 mL of deionized water, followed by the introduction of pretreated nickel foam (1*2 cm^2^). The mixture was then maintained in a shaking incubator at 50°C for 2 h. Subsequently, RuCl_3_·3H_2_O was dissolved in 200 µL of 1 mol L^−1^ HCl, ultrasonically mixed to obtain a homogeneous solution, and then added to the above reaction system for further reaction for 4 h. After completion, the obtained samples were repeatedly washed with deionized water and ethanol and then naturally dried. It should be noted that the NF partially reacts with the acidic solution during the synthesis process. The electrocatalyst precursors were heated in a muffle furnace with a heating rate of 2°C min^−1^ from room temperature to 300°C for 2 h, yielding NiFeRuO_x_ (1:1), NiFe_0.5_RuO_x_ (1:2), NiFe_0.33_RuO_x_ (1:3), and NiFe_0.25_RuO_x_ (1:4) electrocatalysts. Using sodium hypophosphite as the phosphorus source, the catalysts were placed in a tubular furnace under a nitrogen atmosphere, heated at a rate of 2°C min^−1^ to 300°C, and calcined for 2 h to obtain NiFe_0.33_RuO_x_@P.

To clarify the potential contribution of the NF substrate and its acid‐induced modification, control experiments were conducted using pristine Ni foam and HCl‐treated Ni foam (without catalyst deposition). As shown in Figure S13, both samples exhibit significantly inferior HER activity compared to NiFe_0.33_RuO_x_@P. Although HCl treatment slightly improves the activity of Ni foam, the enhancement is limited. These results indicate that the superior HER performance is primarily derived from the NiFe_0.33_RuO_x_@P active layer, while the nickel foam mainly serves as a conductive substrate with a negligible catalytic contribution.

### 4.3 Performance Testing

The HER performance was evaluated via LSV using a standard three‐electrode system in 1.0 m KOH electrolyte (pH = 14), with Hg/HgO as the reference electrode and a graphite rod counter electrode. NiFe_y_RuO_x_ and NiFe_0.33_RuO_x_@P served as the working electrode. Commercial Pt/C (20 wt.%) served as the benchmark catalyst. The Tafel slope was obtained from the linear fitting of the LSV curve in the Faradaic region. The equation for calculating the Tafel slope, as shown in Equation (3), can be used to describe the current‐potential relationship.

(3) η=blog|j|+a

where *η* refers to the overpotential, *b* is the Tafel slope, a is the Tafel intercept, and *j* is the current density.

The double‐layer capacitance (C_dl_) was measured by cyclic voltammetry (CV) in the non‐Faradaic potential window of −0.2 to −0.3 V (vs. Hg/HgO) at scan rates between 10 and 120 mV/s. The capacitance was calculated from the linear fitting of △j = (j_a_—j_c_)/2 with respect to the scan rate, where j_a_ and j_c_ are the anodic and cathodic currents at xxx V, respectively. The electrochemical surface area (ECSA) was estimated using equation ECSA = C_dl_/C_s_ with C_s_ = 40 µF·cm^−2^, It should be noted that this value corresponds to a smooth planar surface; thus, the calculated ECSA serves only as a relative comparison rather than an absolute value for the porous Ni foam‐supported electrodes. All potentials were corrected by the 90% iR compensation command. The half‐cell performance characterization was performed using a three‐electrode system, where the sample was used as the working electrode directly clamped by a Pt clip electrode, a Hg/HgO electrode served as the reference electrode, and a carbon rod acted as the counter electrode. Stability tests were performed at current densities of 10, 100, and 500 mA cm^−2^, respectively.

Full‐cell evaluations were conducted in both a 1 and 25 cm^2^ membrane electrode assembly (MEA) electrolyzer under simulated industrial alkaline conditions. For the 1 cm^2^ MEA configuration, the NiFe_0.33_RuO_x_@P was employed as the HER electrode (cathode), while pretreated NF was used as the OER electrode (anode). A polyphenylene sulfide (PPS) membrane was used as the separator between the two electrodes. For comparison, a commercial Pt/C catalyst was used as the HER electrode under identical conditions, with pretreated NF serving as the OER electrode. For the performance evaluation with an electrode area of 25 cm^2^, a similar configuration was adopted, in which the NiFe_0.33_RuO_x_@P was used as the HER electrode and nickel foam as the OER electrode. The electrolyte (6.0 m KOH) was continuously circulated using a peristaltic pump (SHENCHEN LabV6‐III) to ensure stable mass transport and electrolyte homogeneity during operation. The flow rate of the peristaltic pump was maintained at 100 rpm. All electrochemical measurements were carried out with the assistance of a CS2040B Power Booster current amplifier to extend the measurable current range of the electrochemical workstation. The MEA tests were conducted over a range of temperatures, and polarization curves were recorded to evaluate the overall water‐splitting performance.

### 4.4 Catalyst Characterization

All electrochemical characterization tests were carried out using a Corrtest electrochemical workstation (CS350M). The lattice structure of the sample was determined by X‐ray diffraction (XRD; D8 ADVANCE; Bruker) using Cu Kα radiation (λ = 1.5406 Å) at 40 kV and 40 mA, with a scanning range of 10°–90° (2θ) and a step size of 0.02°. X‐ray photoelectron spectroscopy (XPS, AXIS ULTRA DLD, Japan) was performed using a monochromatic Al Kα source (hν = 1486.6 eV), with a survey scan range of 0–1200 eV, and all spectra were calibrated based on the C 1s peak at 284.8 eV. Scanning electron microscopy (SEM; JSM‐6701F) was used to characterize the surface morphology of the samples. Transmission electron microscopy (TEM; JEOL‐F200) was employed to investigate the microstructure of the sample, and the corresponding energy‐dispersive X‐ray spectroscopy (EDS) mappings were also collected on the same instrument. Quantitative analysis of metal elements was conducted using inductively coupled plasma optical emission spectroscopy (ICP‐OES, Optima 8000), operated with a radio frequency of 27.12 MHz and a spectral detection range of 130–770 nm.

### 4.5 Theoretical Calculation

All calculations in this work were performed based on density functional theory (DFT) using the DMol3 module. The exchange–correlation interactions were described by the generalized gradient approximation (GGA) with the Perdew–Burke–Ernzerhof (PBE) functional. Core electrons were treated using density functional semi‐core pseudopotentials (DSPP), and the valence electrons were expanded with a double numerical plus polarization (DNP) basis set.

The Brillouin zone was sampled using a 7 × 7 × 1 k‐point mesh generated by the Monkhorst‐Pack scheme. A smearing method was applied with a smearing value of 0.005 Hartree to ensure electronic convergence. During geometry optimization, the convergence criteria for total energy, maximum force, and maximum displacement were set to 1.0 × 10^−5^ Ha, 0.002 Ha/Å, and 0.005 Å, respectively.

## Author Contributions

**Yang Li**: conceptualization, methodology, formal analysis, writing – original draft, data curation. **Biao Wang**: investigation, validation, supervision. **Bingbing Niu**: funding acquisition, visualization, project administration, writing – review and editing, conceptualization. **Tao Feng**: writing – review and editing, investigation, validation, conceptualization, supervision. **Dongchao Qiu**: software, validation. **Chunling Lu**: validation, formal analysis.

## Conflicts of Interest

The authors declare no conflicts of interest.

## Supporting information




**Supporting file**: advs76083‐sup‐0001‐SuppMat.docx

## Data Availability

The data that support the findings of this study are available from the corresponding author upon reasonable request.
