# Supplementary material for: A High‐Current‐Tolerant Multimetallic Phosphide Electrode for Alkaline Water Electrolysis Toward Industrial Conditions
Source: Adv Sci (Weinh). 2026 Jun 12:e76083. Online ahead of print. doi: 10.1002/advs.76083 (PMC13336816; doi:10.1002/advs.76083)
Supplement: Supplementary file 1 — Supporting file: advs76083‐sup‐0001‐SuppMat.docx [file ADVS-9999-e76083-s001.docx]

Supporting Information

**A High-Current-Tolerant Multimetallic Phosphide Electrode for Alkaline Water Electrolysis Toward Industrial Conditions**

Yang Li^a^, Chunling Lu^a^, Biao Wang^a^, Dongchao Qiu^a^, Bingbing Niu^a,^^b^[[1]](#footnote-0)^*^, Tao Feng^^[[2]](#footnote-1)^*c^

*^a^ School of Science, University of Science and Technology Liaoning, Anshan 114051, China*

*^b^ Key Laboratory of Interfacial Physics and Technology, Shanghai Institute of Applied Physics, Chinese Academy of Sciences, Shanghai 201800, China*

*^c^ Department of Materials Science and Engineering, Southern University of Science and Technology, Shenzhen 518055, China*

**DFT model：**

To elucidate the evolution of the electronic structure and hydrogen adsorption behavior of the catalyst before and after phosphorization, density functional theory models were constructed for both NiFe_0.33_RuO_x_@P and NiFe_0.33_RuO_x_ (Figure S11 and S12). As illustrated in the structural models, a NiO crystal was first selected as the parent framework. Fe and Ru atoms were then introduced into the supercell to simulate the multimetallic coordination environment in the experimental catalyst. Considering the relatively low content and dispersed distribution of Fe and Ru in the real sample, these two elements were modeled by partially substituting Ni sites, thereby generating the pristine NiFe_0.33_RuO_x_​structure. In this model, the oxide framework preserves the dominant Ni-O coordination, while localized Fe-O and Ru-O environments are formed to represent the multimetal active centers in the oxide precursor.

Based on this structure, the phosphorized NiFe_0.33_RuO_x_@P model was further established. According to the experimental characterization and the actual structural models, P atoms were preferentially introduced near the surface metal sites and coordinated with the outermost metal atoms to form M-P bonds (M = Ni, Fe, and Ru), thereby simulating the metal-phosphorus coupling generated during phosphorization. It should be noted that the phosphorized structure was not treated as an ideal fully converted phosphide phase. Instead, part of the original oxygen coordination was retained, while localized M-P bonding motifs were introduced on the surface, making the model more consistent with the experimentally observed phosphide/oxide coexistence.


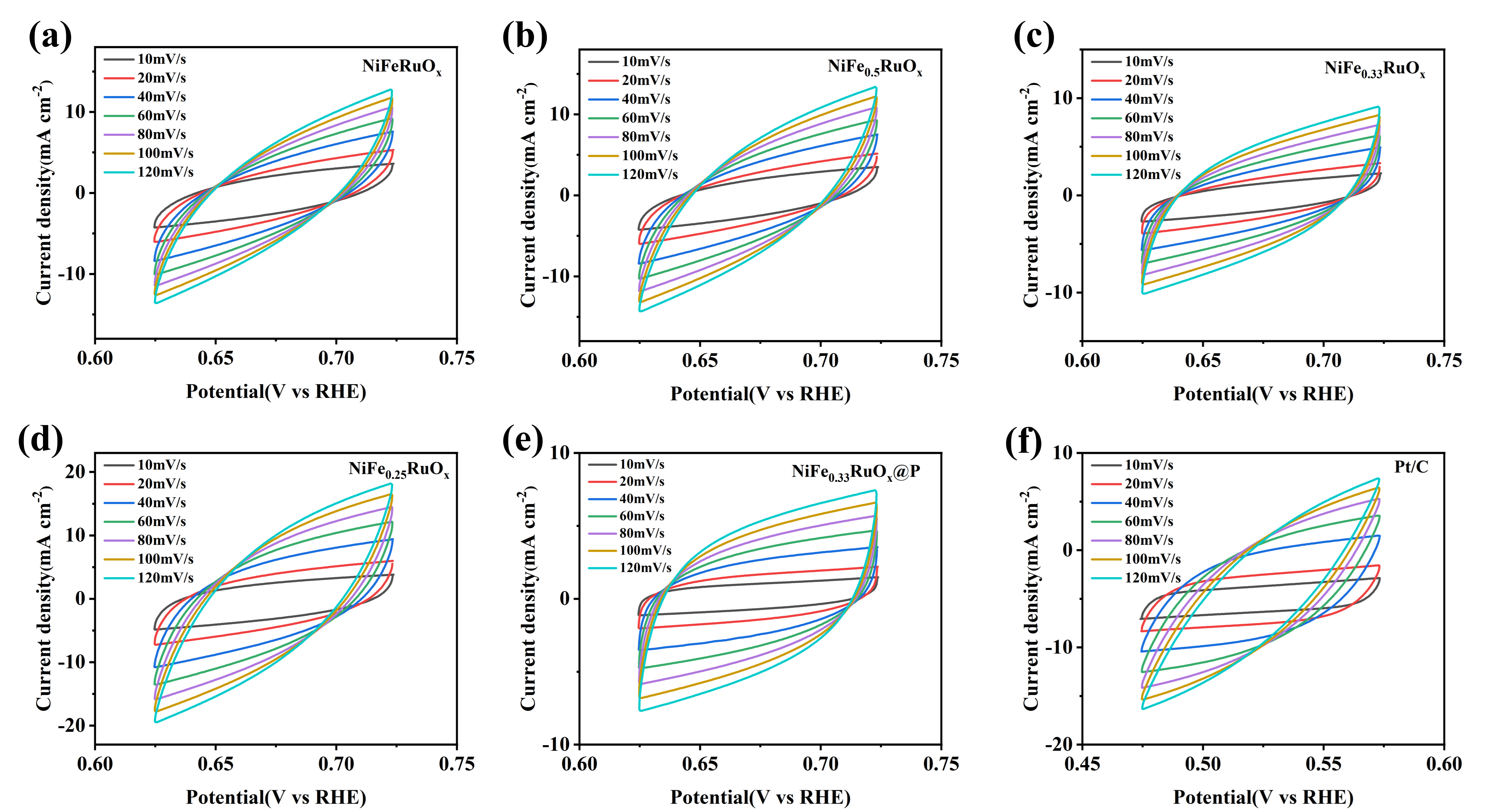


**Figure S1.** (a-f) The CV curve of NiFe_y_RuO_x_, NiFe_0.33_RuO_x_@P and Pt/C in 1.0 M KOH at sweep rate ranging from 10 to 120 mV/s.


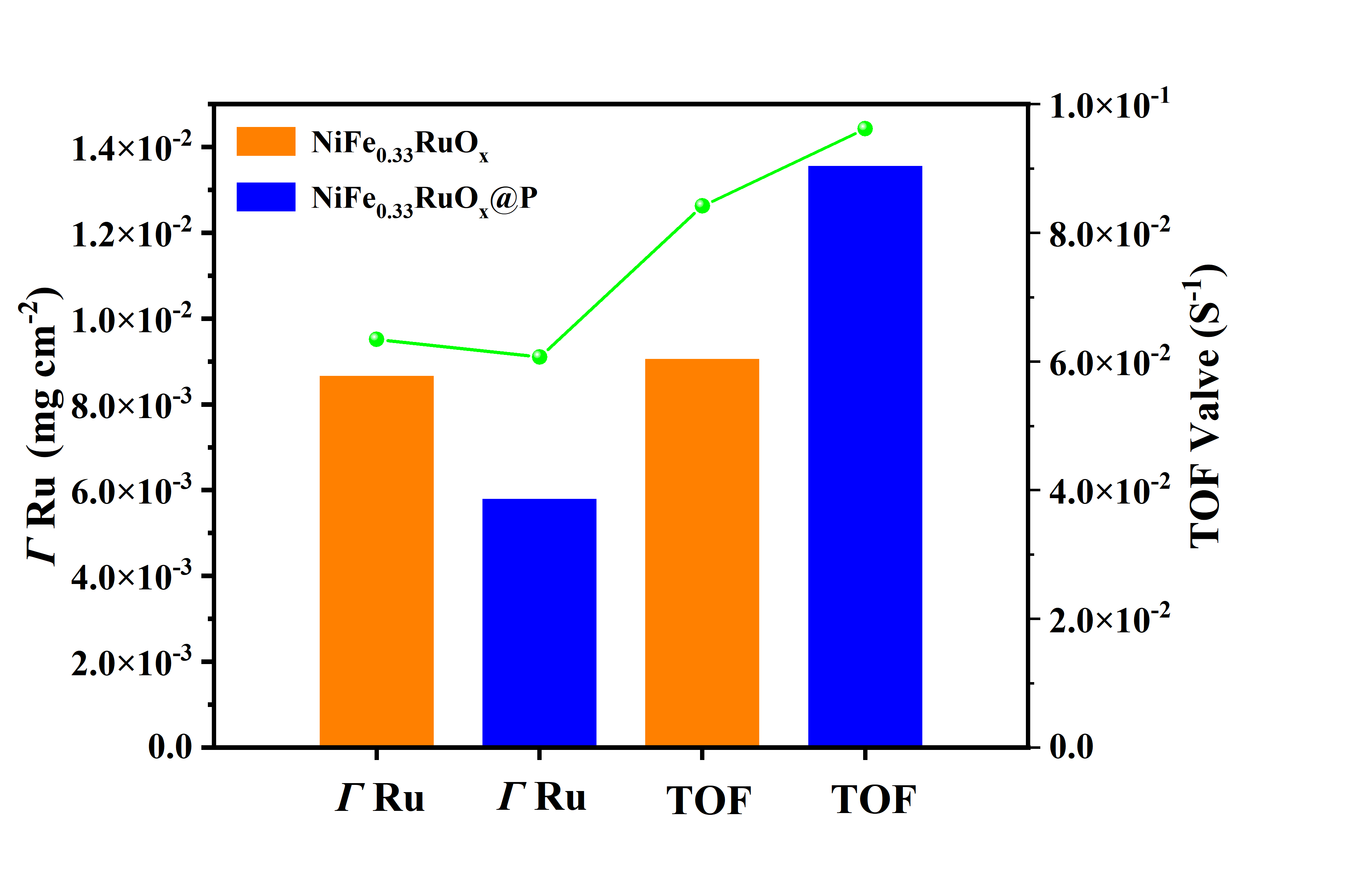


**Figure S2.** Comparison of the Ru areal loading ($\Gamma_{Ru}$) and turnover frequency (TOF) for NiFe_0.33_RuO_x_ and NiFe_0.33_RuO_x_@P at a current density of 100 mA cm^-2^.


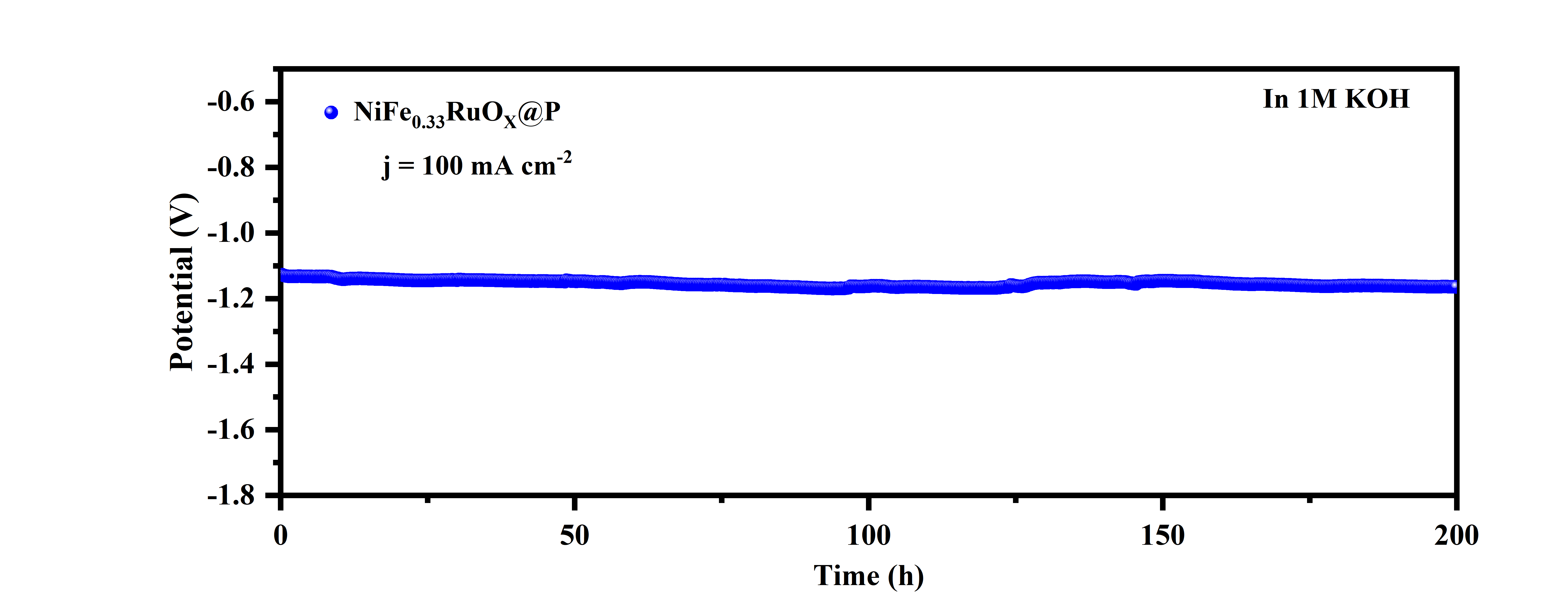


**Figure S3.** Three-electrode stability test for HER of NiFe_0.33_RuO_x_@P at -100 mA cm^-2^ for 200h.

*
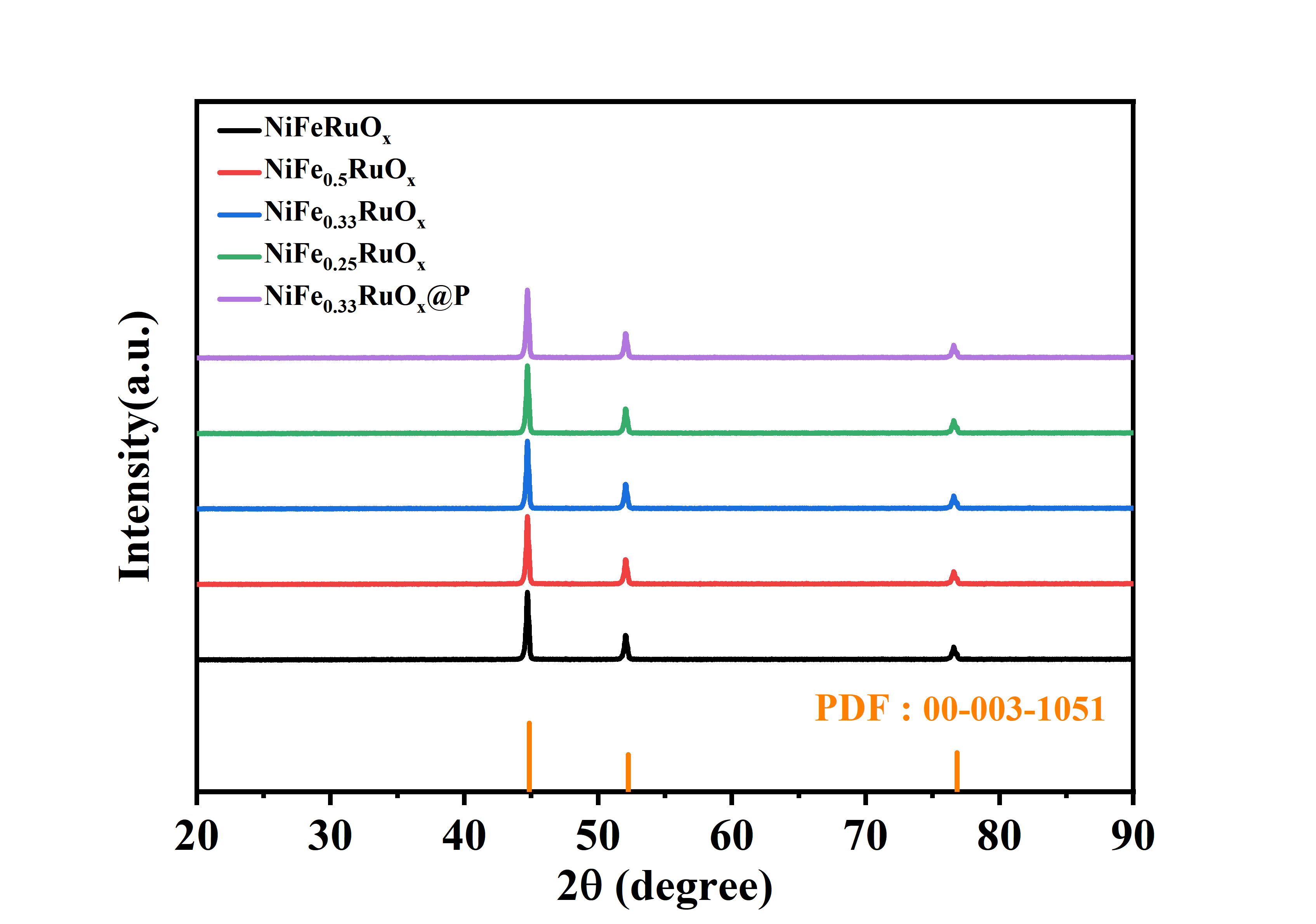
*

**Figure S4.** The XRD image of NiFe_y_RuO_x_ and NiFe_0.33_RuO_x_@P.


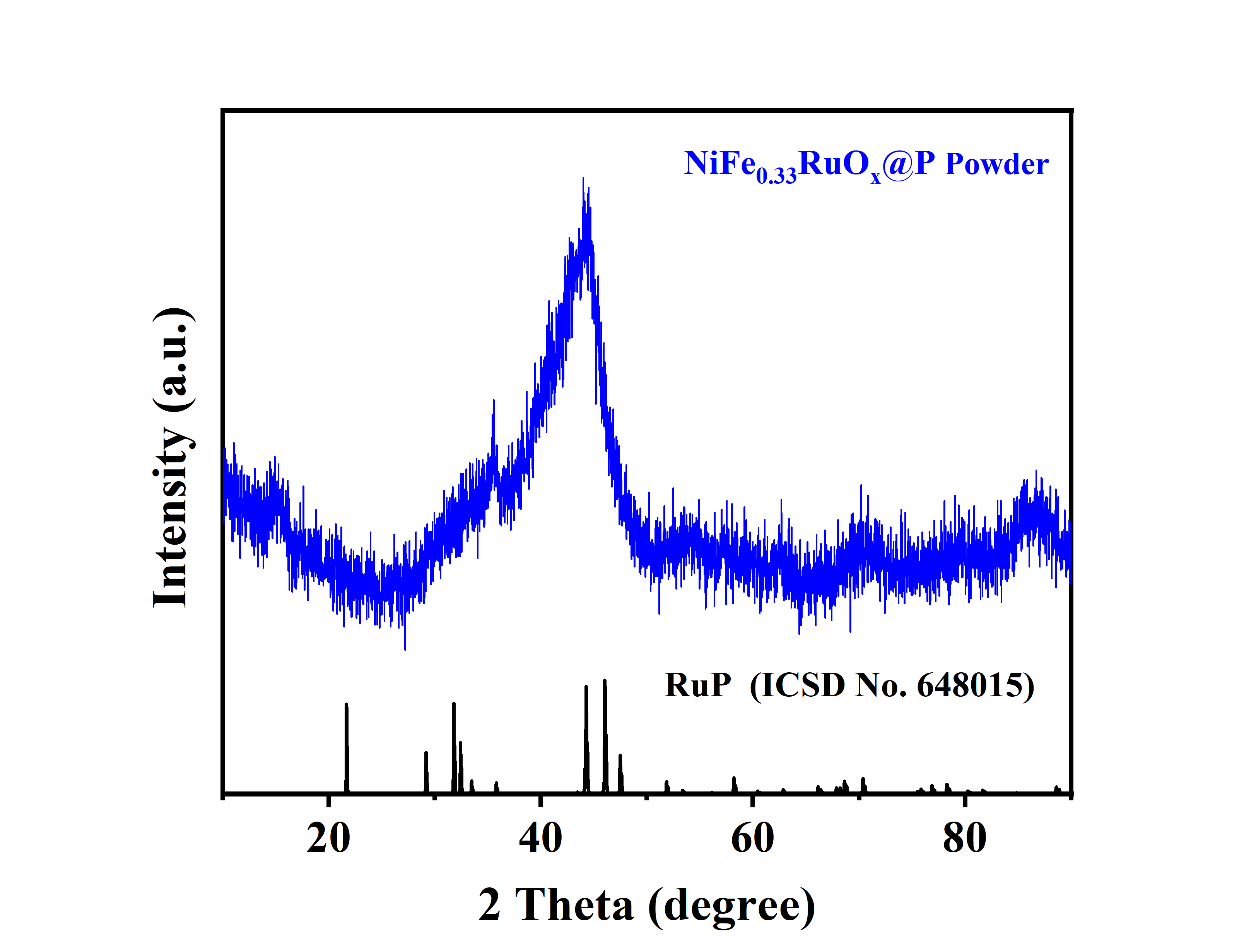


**Figure S5.** The XRD result of NiFe_0.33_RuO_x_@P powder.

**
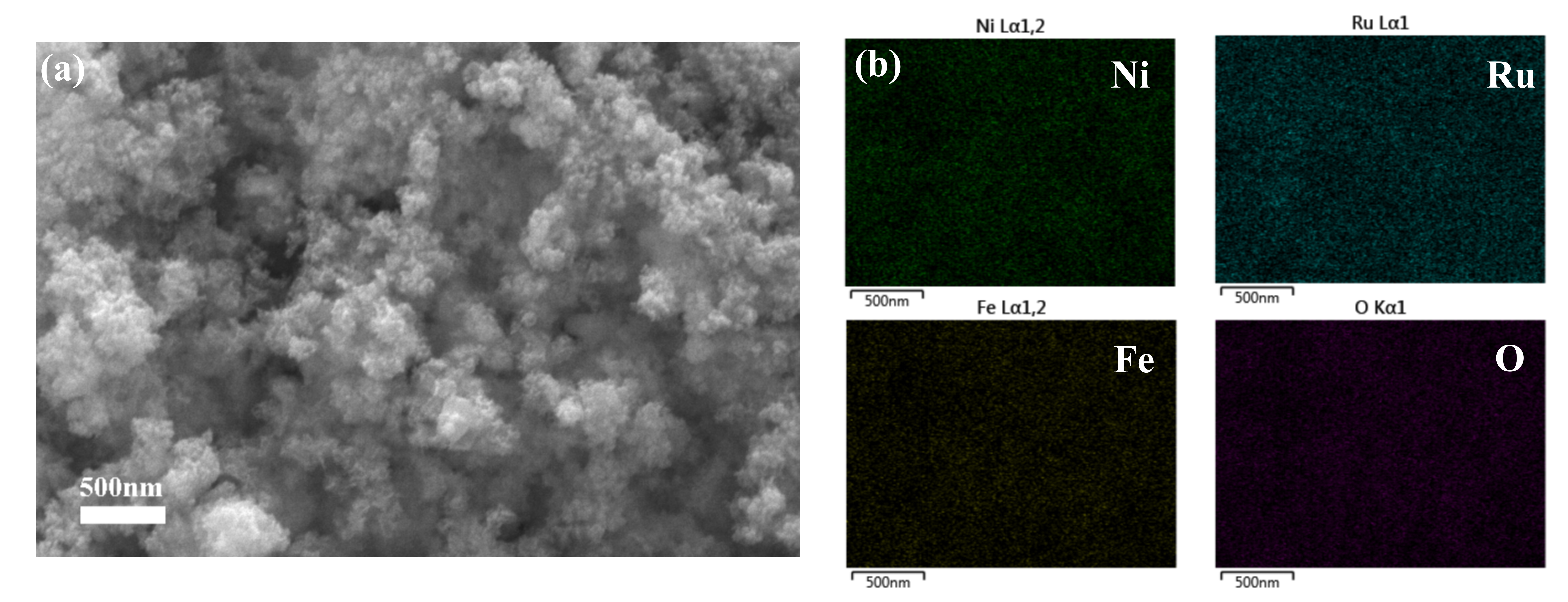
**

**Figure S6.** (a) SEM image and (b) EDS elemental mapping images of the NiFe_0.33_RuO_x_.

*
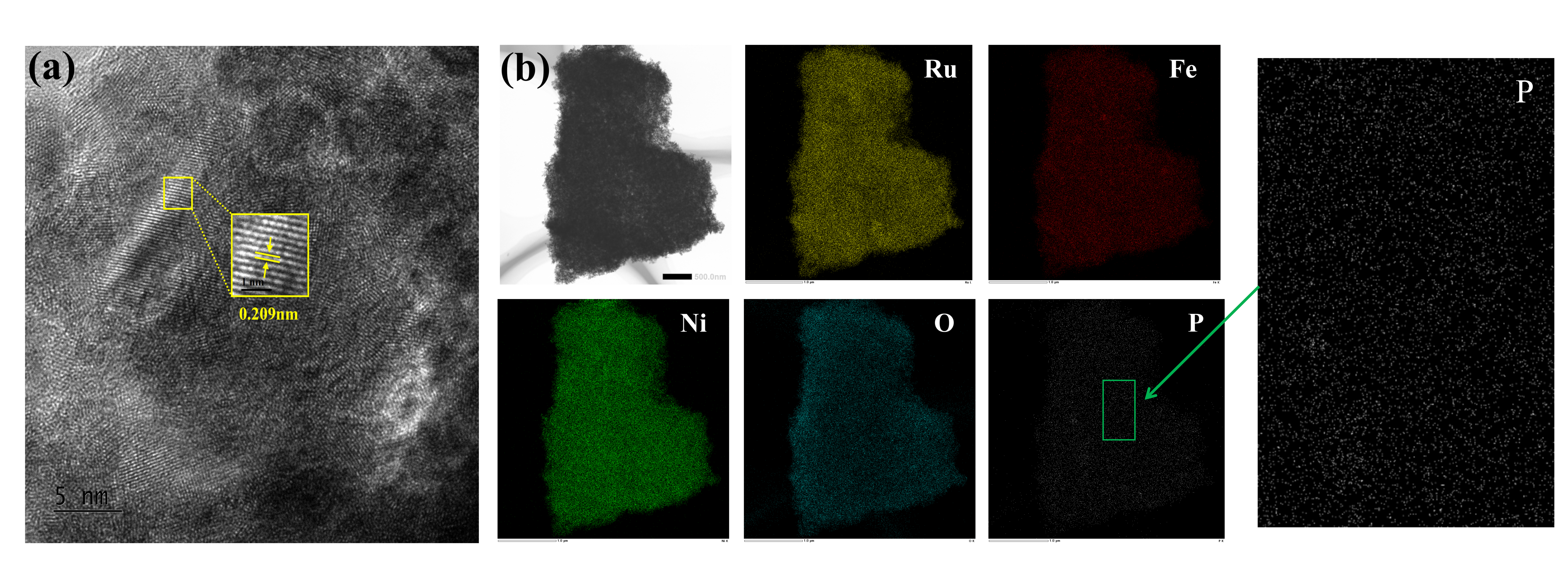
*

**Figure S7.** (a) The HRTEM image of NiFe_0.33_RuO_x_@P. (b) The STEM-EDS of NiFe_0.33_RuO_x_@P.


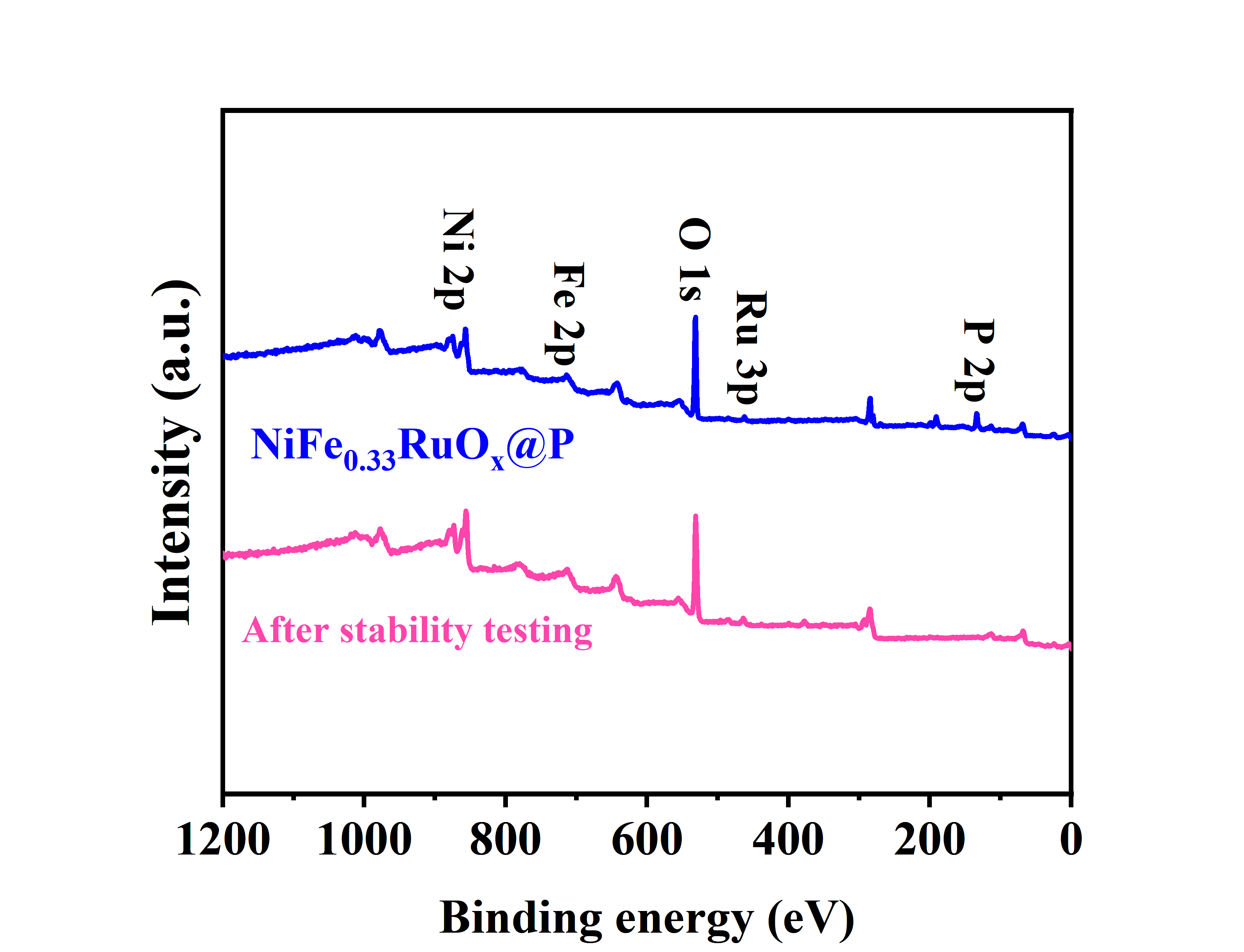
**Figure S8.** XPS full survey spectra before and after stability testing.


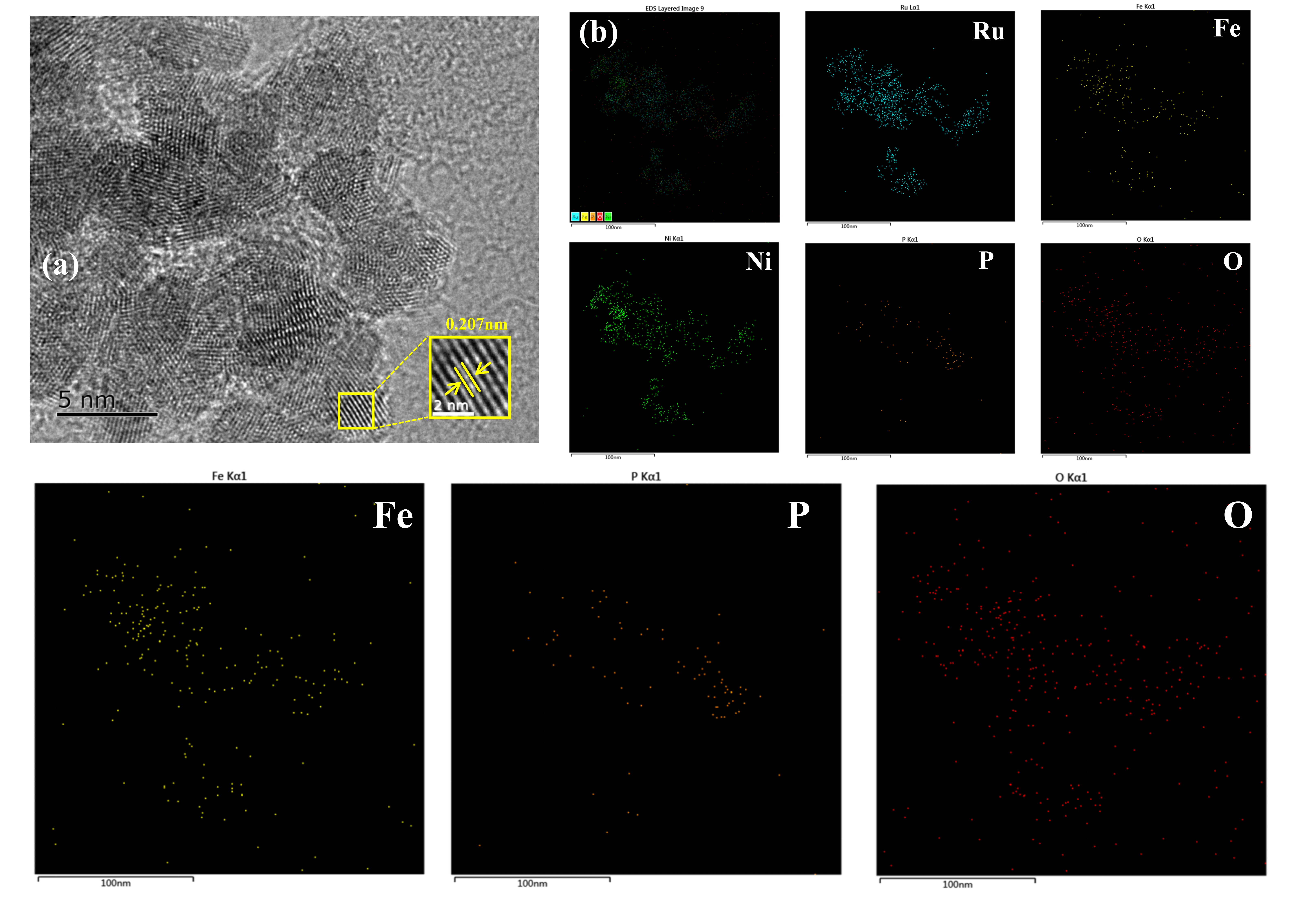
**Figure S9.** (a) The HRTEM image of NiFe_0.33_RuO_x_@P after stability testing . (b) The STEM-EDS of NiFe_0.33_RuO_x_@P after stability testing.


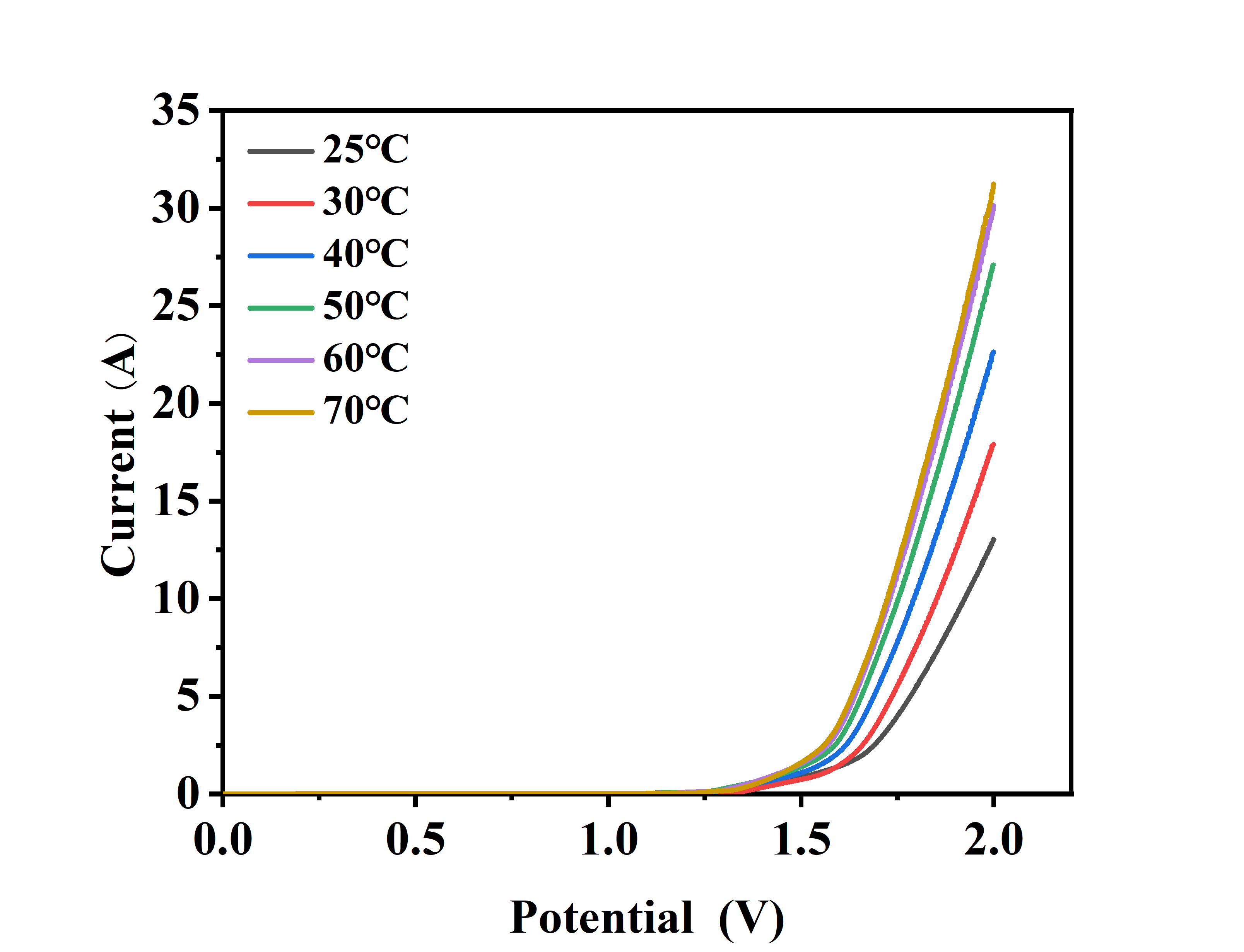
**Figure S10.** LSV curves of NiFe_0.33_RuO_x_@P with an electrode area of 25 cm^2^ measured at different temperatures.

**
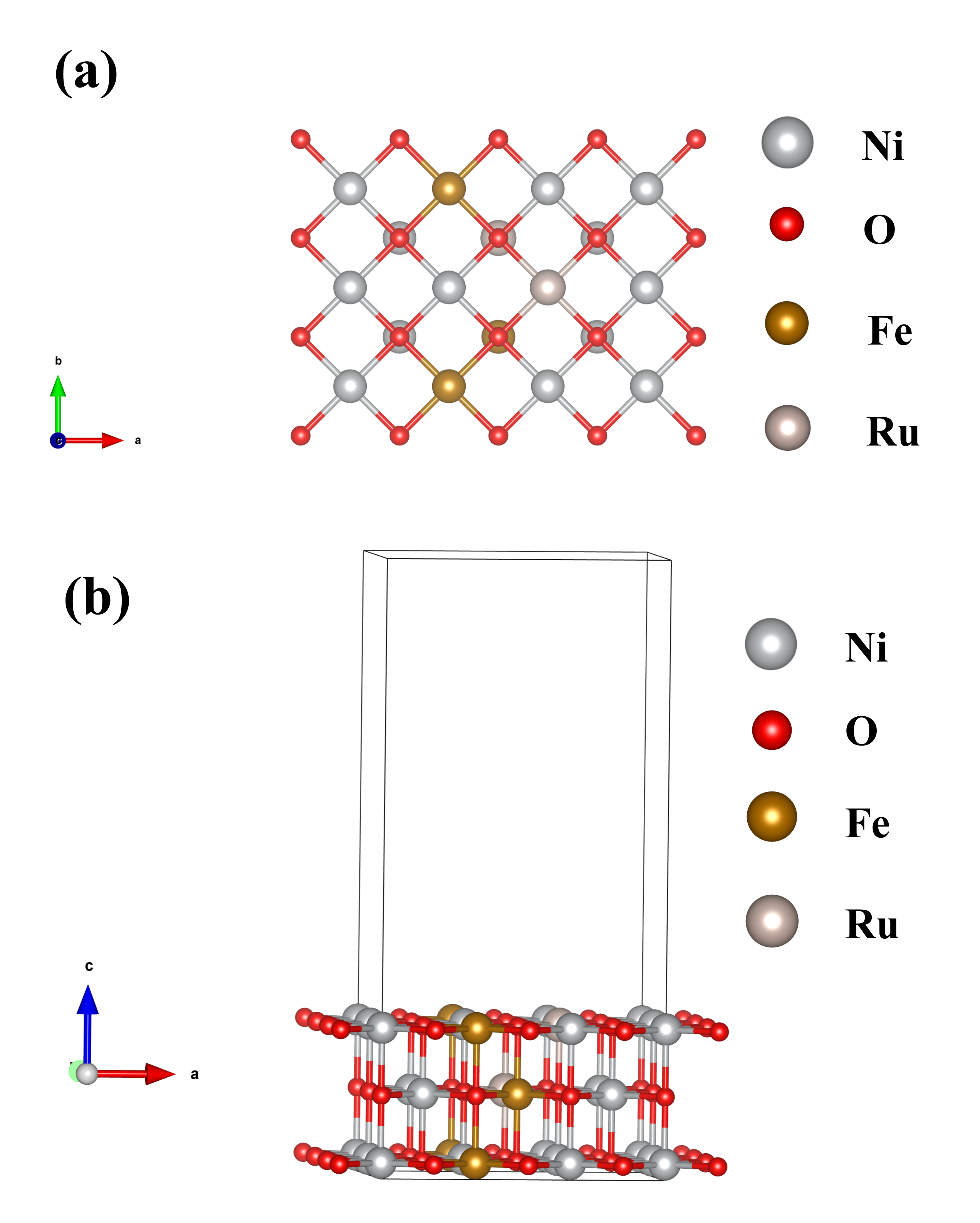
Figure S11.** (a) Optimized atomic structure of the NiFe_0.33_RuO_x_ model used for density functional theory calculations. (b) Optimized surface configuration of NiFe_0.33_RuO_x_.

**
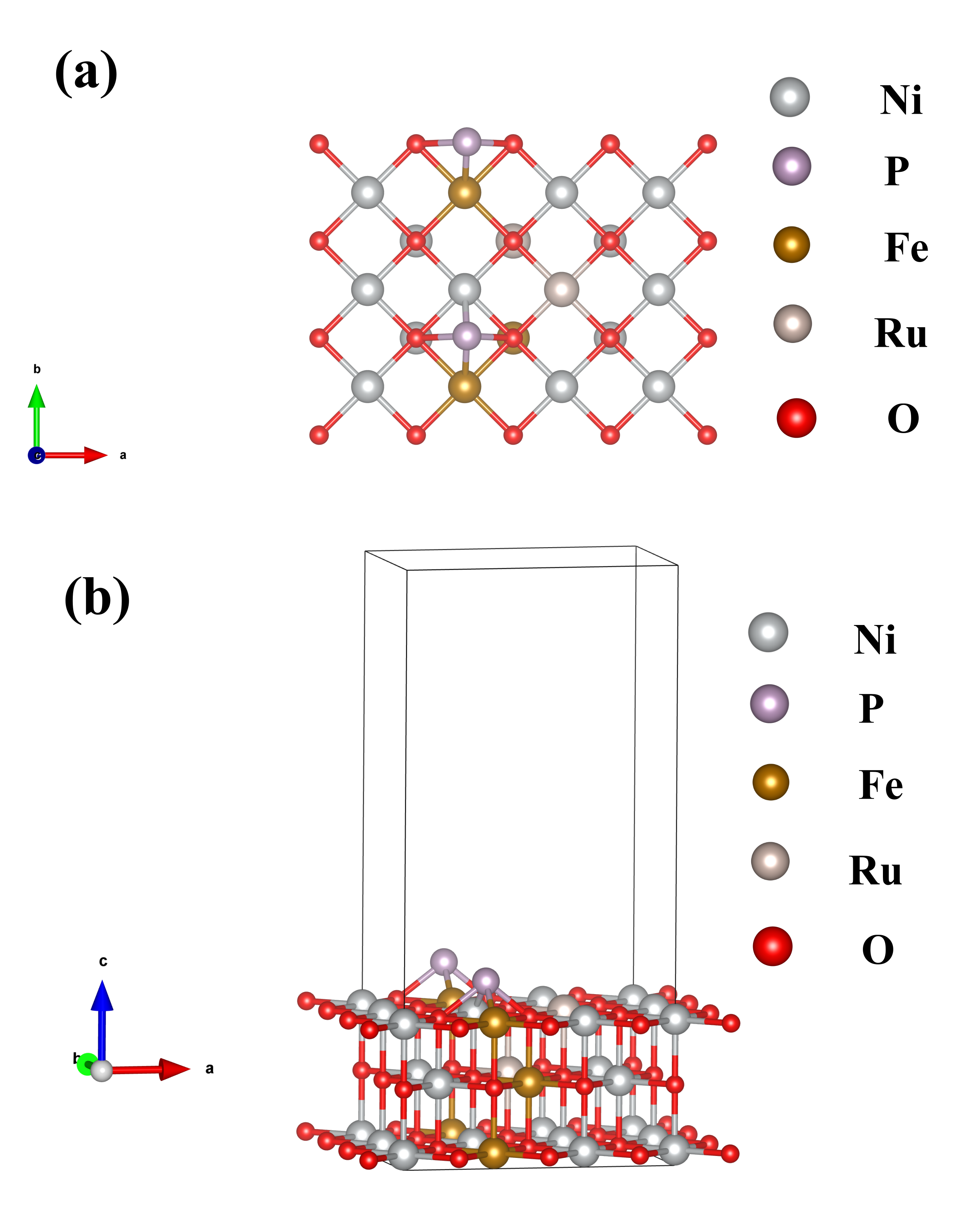
Figure S12** (a) Optimized atomic structure of the NiFe_0.33_RuO_x_@P model used for density functional theory calculations. (b) Optimized surface configuration of NiFe_0.33_RuO_x_@P.


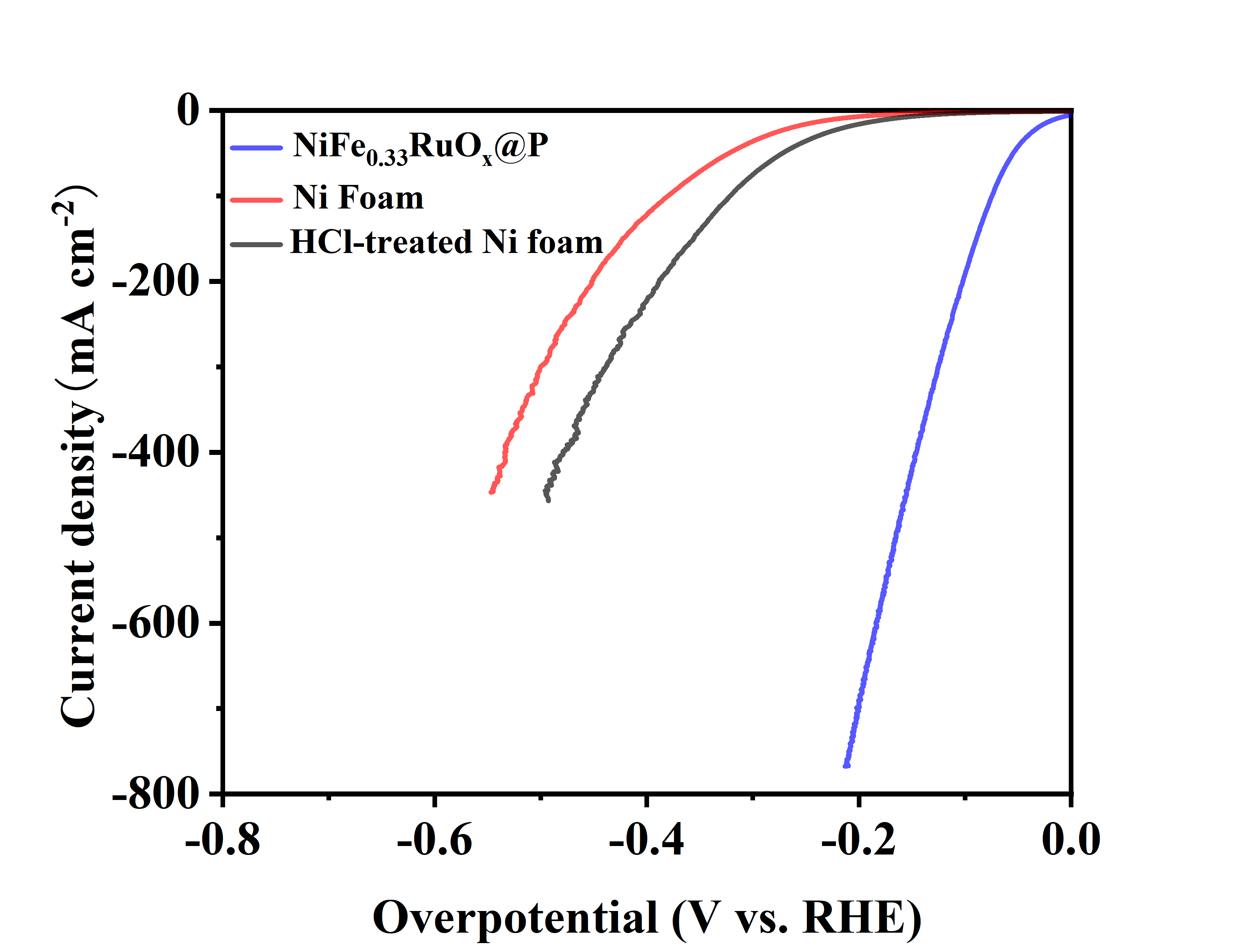


**Figure S13.** The comparison of HER performance of NiFe_0.33_RuO_x_@P and Ni Foam and HCl-treated Ni foam.

**Table S1.** Comparison of HER performance with the electrocatalysts at 100 mA cm**^-^**^2^ current density, Tafel slope in 1 M KOH, and their stability.

| **Electrocatalysts** | **Overpotential at 100 mA cm^-2^** | **Tafel Slope** | **Stability** | **Reference** |
| --- | --- | --- | --- | --- |
| **NiFe_0.33_RuO_x_@P** | **72 mV** | **36.4 mV dec^-1^** | **@-500 mA cm^-2^**  **200 h** | **This work** |
| Ru@WNO-C | 144 mV | 38.9 mV dec^-1^ | 10000 cycles | ^[1]^ |
| Ru_0.2_CoFeP/NF | 78 mV | 30.8 mV dec^-1^ | @-100 mA cm^-2^  120 h | ^[2]^ |
| Ru_1_-NiCoCuMo HEAs | 71 mV | 76.2 mV dec^-1^ | @-100 mA cm^-2^ 800 h | ^[3]^ |
| Ru-O-Ru clusters | 86 mV | 29.2 mV dec^-1^ | @-100 mA cm^-2^  50 h | ^[4]^ |
| Ru-NiFe-③/NF | 90 mV | 42.7 mV dec^-1^ | @-50 mA cm^-2^  20 h | ^[5]^ |
| CoFeP@C/NF | 104 mV | 23.47 mV dec^-1^ | @-50 mA cm^-2^  60 h | ^[6]^ |
| Ni-Ru@Fe/C@CNT | 110 mV | 54.0 mV dec^-1^ | @-0.1 V cm^-2^  25 h | ^[7]^ |
| Ru-Zn/MWCNTs | 119 mV | 44.5 mV dec^-1^ | 10000 cycles | ^[8]^ |
| Ru-doped NiO/Co_3_O_4_ | 138 mV | 58.0 mV dec^-1^ | @-120 mA cm^-2^ 25 h | ^[9]^ |
| Ru/V_2_O_3_-CC | 219 mV | 45.9 mV dec^-1^ | @-100 mA cm^-2^  20 h | ^[10]^ |
| NiRuO_x_-Ar | 94 mV | 52.73 mV dec^-1^ | @-50 mA cm^-2^  100 h | ^[11]^ |
| Ru-Co@Ti_2_AlC | 95 mV | 105 mV dec^-1^ | @-20 mA cm^-2^  48 h | ^[12]^ |
| Ru-NiO/CNTs | 98 mV | 56.5 mV dec^-1^ | @-10 mA cm^-2^  100 h | ^[13]^ |
| RuO_2_−Ti_3_C_2_/NF | 85 mV | 127.5 mV dec^-1^ | @-20 mA cm^-2^  40 h | ^[14]^ |

**Reference**

1. G. Meng, H. Tian, L. Peng, et al., “Ru to W electron donation for boosted HER from acidic to alkaline on Ru/WNO sponges,” *Nano Energy* 80, no. (2021), <https://doi.org/10.1016/j.nanoen.2020.105531>.

2. D. Guo, X. Guo, L. Wen, and X. Li, “Ru-doped cobalt-iron bimetallic phosphide nanoflowers: Electronic structure modulation for high-efficiency hydrogen evolution reaction,” *J Colloid Interface Sci* 699, no. Pt 1 (2025): 138116, <https://doi.org/10.1016/j.jcis.2025.138116>.

3. Y. Zou, H. Zhao, W.-D. Zhang, et al., “High entropy alloy supported ruthenium single-atoms for enhanced electrochemical hydrogen evolution reaction,” *Chemical Engineering Journal* 518, no. (2025), <https://doi.org/10.1016/j.cej.2025.164784>.

4. D. Liu, L. Xu, S. Li, et al., “Atomically precise Ru-O-Ru clusters for enhanced water dissociation in alkaline hydrogen evolution,” *Nano Research* 17, no. 8 (2024): 6993-7000, <https://doi.org/10.1007/s12274-024-6726-y>.

5. Y. Wang, C. Wang, H. Shang, et al., “Self-driven Ru-modified NiFe MOF nanosheet as multifunctional electrocatalyst for boosting water and urea electrolysis,” *J Colloid Interface Sci* 605, no. (2022): 779-789, <https://doi.org/10.1016/j.jcis.2021.07.124>.

6. J. Ren, J. Liu, Y. Du, et al., “Trace ruthenium promoted dual-reconstruction of CoFeP@C/NF for activating overall water splitting performance beyond precious-metals,” *Nano Research* 16, no. 8 (2023): 10810-10821, <https://doi.org/10.1007/s12274-023-5825-5>.

7. T. Gao, X. Li, X. Chen, et al., “Ultra-fast preparing carbon nanotube-supported trimetallic Ni, Ru, Fe heterostructures as robust bifunctional electrocatalysts for overall water splitting,” *Chemical Engineering Journal* 424, no. (2021), <https://doi.org/10.1016/j.cej.2021.130416>.

8. H. Zhang, S. Qi, K. Zhu, and X. Zong, “Ruthenium nanoclusters modified by zinc species towards enhanced electrochemical hydrogen evolution reaction,” *Front Chem* 11, no. (2023): 1189450, <https://doi.org/10.3389/fchem.2023.1189450>.

9. J. Zhang, J. Lian, Q. Jiang, and G. Wang, “Boosting the OER/ORR/HER activity of Ru-doped Ni/Co oxides heterostructure,” *Chemical Engineering Journal* 439, no. (2022), <https://doi.org/10.1016/j.cej.2022.135634>.

10. X.-Z. Fan, Q.-Q. Pang, F. Fan, H.-C. Yao, and Z.-J. Li, “Ultra-fine Ru nanoparticles decorated V2O3 as a pH-universal electrocatalyst for efficient hydrogen evolution reaction,” *International Journal of Hydrogen Energy* 48, no. 54 (2023): 20577-20587, <https://doi.org/10.1016/j.ijhydene.2023.03.046>.

11. N. Xu, M.-M. Li, H.-Y. Sun, et al., “Atmosphere-driven oriental regulation of Ru valence for boosting alkaline water electrolysis,” *Chemical Engineering Journal* 502, no. (2024), <https://doi.org/10.1016/j.cej.2024.158051>.

12. D. Kutyła, M. N. Krstajić Pajić, U. Č. Lačnjevac, M. M. Marzec, N. R. Elezović, and P. Żabiński, “Ru–Co alloy coatings electrodeposited on a MAX phase substrate as efficient catalysts for the hydrogen evolution reaction,” *International Journal of Hydrogen Energy* 56, no. (2024): 28-40, <https://doi.org/10.1016/j.ijhydene.2023.11.296>.

13. Y. Chen, Y. Lee, W. Chu, and J. Li, “Trace Ru-tuned NiO/CNT electrocatalysts outperform benchmark Pt for alkaline hydrogen evolution with superior mass activity,” *Chemical Engineering Journal* 472, no. (2023), <https://doi.org/10.1016/j.cej.2023.144922>.

14. Y. Zhang, Z. Zhang, Z. Yu, et al., “Ruthenium Oxide Nanoparticles Immobilized on Ti3C2 MXene Nanosheets for Boosting Seawater Electrolysis,” *ACS Applied Materials & Interfaces* 15, no. 50 (2023): 58345-58355, <https://doi.org/10.1021/acsami.3c12254>.

1. * Corresponding author. E-mail: [niubb92@foxmail.com](mailto:niubb92@foxmail.com) (B. Niu) [↑](#footnote-ref-0)
2. * Corresponding author. E-mail: [fengt@sustech.edu.cn](mailto:fengt@sustech.edu.cn) (T. Feng) [↑](#footnote-ref-1)
